# Visual adaptation and face perception

**DOI:** 10.1098/rstb.2010.0360

**Published:** 2011-06-12

**Authors:** Michael A. Webster, Donald I. A. MacLeod

**Affiliations:** 1 Department of Psychology, University of Nevada, Reno, NV 89557, USA; 2 Department of Psychology, University of California, San Diego, La Jolla, CA 92093, USA

**Keywords:** face perception, adaptation, colour vision, after-effects, neural coding

## Abstract

The appearance of faces can be strongly affected by the characteristics of faces viewed previously. These perceptual after-effects reflect processes of sensory adaptation that are found throughout the visual system, but which have been considered only relatively recently in the context of higher level perceptual judgements. In this review, we explore the consequences of adaptation for human face perception, and the implications of adaptation for understanding the neural-coding schemes underlying the visual representation of faces. The properties of face after-effects suggest that they, in part, reflect response changes at high and possibly face-specific levels of visual processing. Yet, the form of the after-effects and the norm-based codes that they point to show many parallels with the adaptations and functional organization that are thought to underlie the encoding of perceptual attributes like colour. The nature and basis for human colour vision have been studied extensively, and we draw on ideas and principles that have been developed to account for norms and normalization in colour vision to consider potential similarities and differences in the representation and adaptation of faces.

## 1. Visual adaptation and the perception of faces

**Figure 1.**
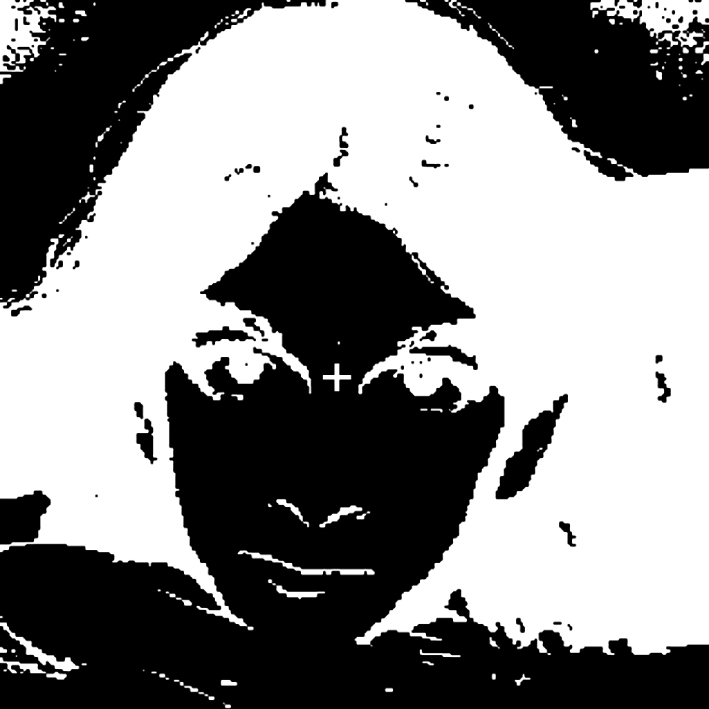
An illustration of a negative after-effect from light adaptation.

Stare carefully at the cross in the image in figure 1 for a minute or so, and then close your eyes. A clear image of a face will appear after a few seconds. (A popular version of this illusion uses an image of Christ, so we have tried it with a devil.) The phantom image is a negative afterimage of the original picture, and arises because each part of the retina adjusts its sensitivity to the local light level in the original picture. Thus, when a uniform field of dim light is transmitted by the closed eyelids, cells that were previously exposed to dark (or light) regions will respond more (or less). Note that the afterimage is also much more recognizable as a face compared with the original, in part because it has the correct brightness polarity (e.g. dark eyes). This polarity-specific difference [1] is one of many examples that have been used to argue that face perception may depend on specialized processes that are distinct from the mechanisms mediating object recognition [2]. However, while the visual coding of faces may depend on face-specific pathways, the principles underlying how these processes are organized and calibrated may be very general. In particular, the principles of sensitivity regulation that give rise to light adaptation in the retina are likely to be manifest at all stages of visual coding, and may be fundamentally important to all perceptual analyses. Thus, just as adapting to different light levels can affect the perception of brightness, adaptation to different faces can affect the appearance of facial attributes, and in both cases these sensitivity changes may shape the nature of visual coding in functionally similar ways.

In this review, we consider the properties and implications of adaptation effects in face perception. Although it may not be obvious, the appearance of faces does depend strongly on the viewing context, and thus the same face can look dramatically different depending on which faces an observer is previously exposed to. These face after-effects offer a window into the processes and dynamics of face perception and have now been studied extensively. Here, we consider what these studies have revealed about human face perception. But before examining this, it is worth emphasizing that it is important to understand how face perception and recognition are affected by perceptual adaptation for a number of reasons. First, faces are arguably the most important social stimuli for humans and the primary means by which we perceive the identity and state of conspecifics. Any process that modulates that perception thus has profound implications for understanding human perception and behaviour, and these consequences are important regardless of the basis of the adaptation. That is, how our judgements of such fundamental attributes as identity, expression, fitness or beauty are affected by the diet of faces we encounter is essential to understanding the psychology of face perception, whether those context effects result from changes in sensitivity or criterion and whether they reflect high-level and face-specific representations or low-level and thus generic response changes in the visual system. Second, a central focus of research in face perception has been to understand how information about faces is encoded by the visual system, and to what extent this code might be specific for faces. Adaptation provides a potential tool for dissecting this neural code and a means to test whether the neural strategies that have been identified at early levels of the visual pathway can also predict the characteristics of high-level vision. To this end, we compare the after-effects for faces and consider their implications for the format of the neural code by which faces are represented, noting similarities and differences between face adaptation and analogous adaptation effects in human colour vision, where the format of the neural representation is relatively well defined. Finally, face after-effects also provide an important context for studying the processes of adaptation itself; whether the plasticity that is so readily demonstrated for simple sensory attributes is again a general feature of visual coding; and how this plasticity is manifest for the kinds of natural and ecologically important stimuli that the visual system is ultimately trying to digest.

## 2. Adaptation and visual coding

The perceived size and shape of objects can be strongly biased by adaptation. For example, after viewing a tilted line, a vertical line appears tilted in the opposite direction [3], while adapting to a vertically oriented ellipse causes a circle to appear elongated along its horizontal axis [4]. Like light or colour adaptation, these are negative after-effects because the neutral test stimulus appears less like the adapting image and thus biased in the complementary direction. And like colour adaptation, these shape or figural after-effects have been widely studied, because they provide clues about how spatial information is encoded by the visual system. In particular, adaptation is one of the primary tools for investigating the ‘channels’ used to represent visual information [5]. At early stages of the visual system, stimuli are encoded by the relative activity in mechanisms that respond to restricted ranges along the stimulus continuum (e.g. tuned for a particular range in orientation, spatial scale or wavelength) [6]. If the channels have joint tuning on multiple parameters, then they are selective to restricted regions in stimulus parameter space. In its simplest conception, adaptation reduces the responses in the mechanisms that respond to the adapting stimulus, and thus biases the distribution of responses to the test stimulus, leading to changes in the detectability and appearance of the test image. Studies of how these adaptation effects transfer from one stimulus to another can therefore reveal properties of the channels such as their number and selectivity, and thus how information is organized at the sites in the visual system that are affected by the adaptation.

Increasingly, this approach is being extended to analyse more high-level processing as well as more ecologically relevant tasks in perception, including the perception of faces [5,7]. Specifically, adaptation is being used to dissect the channels mediating face coding, and to reveal the principles underlying this code [8]. One fundamental issue is how the channels span the space of possible faces [9]. Do they tile the space so that an individual face is represented by a local peak in the distribution of neural responses (an exemplar code similar to how early spatial coding might represent the scale and orientation of local edges)? Do they instead use a relative code to represent how each individual face differs from a prototype (a norm-based code similar to the way hue and saturation might be represented according to how they deviate from white)? Are the channels tuned to the raw physical properties of the image or to the semantic and phenomenally accessible dimensions of a face (e.g. for particular identities or types of individuals)? And do they represent these properties independently or jointly encode the different constellation of characteristics that uniquely define a face (e.g. its age, expression or identity)? Let us see.

## 3. Figural after-effects in face perception

**Figure 2.**
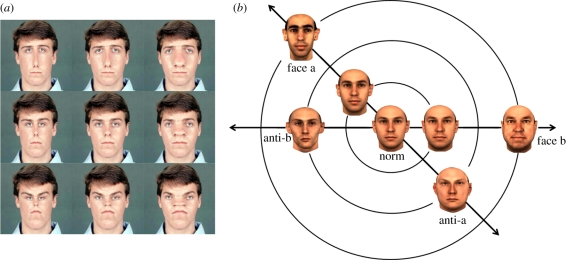
Examples of stimuli used to measure face after-effects. (*a*) A single face distorted by locally stretching or shrinking the face along the vertical or horizontal axis. (*b*) Faces and their ‘antifaces’ formed by projecting the individual face through an average face.

Early signs that adaptation could affect face perception included reports that judgements of facial expression were biased by prior exposure to faces with a different expression [10], and that the aspect ratio of a face appeared altered after seeing face images that were stretched by viewing the faces through cylindrical lenses [11]. Webster & MacLin [12] explored figural after-effects in faces by asking how the appearance of a face changed after adapting to faces that were configurally distorted by locally distending or constricting the image relative to a point on the nose. Figure 2 shows an example of these images formed by different combinations of vertical or horizontal distortions. Exposure to one of the distorted faces (e.g. horizontally constricted) made the original face appear distorted in the opposite way (e.g. horizontally distended). Thus after adapting, the image that appeared most like the original face had to be physically biased towards the adapting distortion in order to compensate for the opposite distortion induced by adaptation. (That is, the negative perceptual after-effect must be cancelled—or nulled—by a positive physical shift towards the adapting image, just as a green after-effect in a previously white light can be cancelled by a physical change towards red.) These after-effects are highly salient in faces and can shift the perceived distortion in the image by 30 per cent or more of the inducing level. They can also strongly bias the set of images that appear normal. For example, after adapting to a distorted image, the range of physical distortions that observers accept as a possible photograph of a face is strongly biased towards the adapting face, again in the direction opposite to the perceptual after-effect [13]. In addition to judging the distortion or normality of the image, the after-effects can also be assessed by judging properties such as the perceived attractiveness of the image [14] and can impact ratings of the personality characteristics of the face [15]. The after-effects have also been assessed with a variety of other measures in addition to nulling. These include recognition thresholds, in which stimulus strength is varied until the individual face can be reliably identified [16]; normality judgements, where the images are classified as depicting a real versus impossibly distorted face [13]; magnitude estimation, where observers use a rating scale to report attributes such as how distorted or attractive a face appears [14]; and contrast detection, in which the after-effects are measured as changes in the luminance contrast required to recognize the face [17].

Leopold *et al*. [16] used a model of real facial variations and observed comparable after-effects when observers were adapted to the configurations corresponding to an individual's face. In their experiments, the stimuli could be varied along ‘identity trajectories’ that passed through the multi-dimensional coordinates defining the individual face and the average face. Increasing distances away from the average towards the individual increased their ‘identity strength’ while stimulus variations in the opposite direction corresponded to an antiface with complementary characteristics. Adaptation to the antiface biased the appearance of the average face so that it appeared more like the target face, and consequently lowered the threshold identity strength required for recognizing the target face.

Adaptations to real facial configurations are sometimes referred to as ‘face identity after-effects’ to distinguish them from the ‘face distortion after-effects’, which are measured by arbitrarily distorting the images [18]. However, it is likely that the underlying processes are intimately linked if not the same, for real configural variations are a form of distortion in the average face, and conversely simple geometric distortions appear as plausible images of real faces. (Indeed, figure 2 suggests that no matter how you distort a normal face, it starts to appear more British.) Moreover, comparable adaptation effects are observed with synthetic faces based on rendering key facial features [19] or face silhouettes [20]. The after-effects also vary somewhat seamlessly between distortions that are within the range of normal variability or are too large to depict a possible human face [13,21,22].

A key implication of the Leopold *et al*. study [16] was that real faces vary in their configural properties enough to and in ways that can induce different states of adaptation. This has been found not only for individual identity but also for the natural variations defining facial categories such as gender and ethnicity [23], as well as natural variations within an individual face such as changes in expression [23,24]. For instance, after adapting to a male face, an androgynous face (formed by morphing between a male and female face) appears more feminine. The adaptation can also influence face perception by changing the appearance of the textural characteristics of the faces rather than their shape [25]. As one example, ageing produces more prominent changes in facial texture than in facial shape, and adaptation to these textural changes can lead to large after-effects in perceived age [26]. Collectively, these results suggest that adaptation is influencing the visual representation of stimulus dimensions that are directly involved in face perception. Perhaps, more importantly, they also suggest that adaptation is probably routinely shaping these representations in normal viewing in ways that are selective for the specific population of faces to which an individual is exposed.

## 4. Dynamics of face adaptation

Do the figural after-effects for faces reflect a common and general process of visual adaptation or are they more akin to other forms of neural plasticity such as perceptual learning or priming? One way this question has been addressed is to examine the time course of the adaptation. Adaptation to simple visual features, in part, reflects transient changes in the visual response which shows a lawful pattern of build-up with increasing adapt time and decay when the adapting stimulus is removed [27]. The build-up and duration of face after-effects follow a very similar time course and functional form to conventional spatial contrast adaptation, suggesting that they involve similar mechanisms [28–30]. However, the dynamics of all visual after-effects are complex and may occur over multiple timescales [31]. Surprisingly, strong face after-effects can be generated following exposures of only a few seconds [16] (a disheartening revelation for those of us who instead spent hours adapting). Extremely brief adapting and test durations have also been found to produce potent after-effects in shape perception and have been used to distinguish global shape after-effects from simpler after-effects produced by features such as local tilt that require longer durations [32]. Similarly, face adaptation may exhibit different properties and potentially different levels of processing at different adapting durations [33]. Like simple after-effects, adaptation to faces can also result in long-term changes, which may be more likely to occur for more familiar faces [34–36]. Distortions in highly familiar faces can lead to particularly noticeable after-effects. In part, this may be because observers are more sensitive to them [37]. However, there is evidence that familiarity also changes the actual strength of the adaptation and how it generalizes across stimulus dimensions such as changes in viewpoint, and thus the properties of the adaptation may also mirror other dynamics of how the visual representation of faces might change with experience [38,39].

## 5. Sites of sensitivity change

Figure 1 is an example of an after-effect that arises very early in the visual system—in the response changes of the photoreceptors—but propagates to levels where the pattern of activity is interpreted as a face. This highlights the point that adaptation can occur throughout the visual pathway, and thus high-level processes can inherit the response changes from earlier levels [40]. There is little doubt that adaptation to a face image induces response changes in visual mechanisms that are sensitive to simple features in the image. A central question is whether, in addition to these low-level response changes, there are also aspects of the adaptation that arise at high and possibly face-specific levels of visual processing.

### (a) Image versus object

One approach to this question has been to ask whether the adaptation is more closely tied to retinocentric or object-centric representations. Compared with low-level after-effects, adaptation to faces shows substantial transfer across changes in size and location [14,16,41–43]. In fact, these size changes are often included as a control to reduce the contribution of early retinotopic response changes. However, the strongest after-effects persist when the adapt and test faces give rise to the same proximal stimulus (i.e. the same retinal image) [44,45]. The broad but incomplete transfer of the adaptation across spatial transformations is consistent with the larger receptive fields and spatial tolerances characterizing extrastriate areas implicated in face perception like the fusiform face area (FFA) [46]. The after-effects also show partial transfer across changes in orientation. In a clever test of this transfer, Watson & Clifford [47] used faces that were distorted along one axis of the face (e.g. vertically). Adapting and test faces were then shown tilted, with an orientation difference of 90° (e.g. adapt tilted 45° clockwise and test tilted 45° counterclockwise). This predicted after-effects that were in different directions depending on whether the response changes are tied to the spatial axis of the adaptation (always 45° clockwise) or to the axis of the object (a vertical distortion within the face regardless of the spatial orientation of the head). The observed after-effects rotated with the object, again implicating a high-level process in the adaptation.

### (b) Inversion effects

The effects of orientation on face adaptation have received considerable attention. Compared with other objects, faces are more difficult to recognize when they are upside down [48], and this is thought to occur because the configural or holistic processing required for sensitive face recognition is lost when the image is inverted [49]. This inversion effect is therefore considered a hallmark of face perception [50]. A number of studies have explored whether the adaptation also shows an inversion effect. Initial tests suggested that it does not. After-effects of comparable strength can occur for upright or inverted faces [12,16,47], or for faces that have the correct or inverted contrast polarity [42]. However, there is little transfer of the after-effect between original and inverted images [12], and in fact opposite after-effects can be induced simultaneously between them [51]. Notably, these inversion-contingent after-effects cannot entirely reflect processing differences between upright and inverted images, because they also occur for pairs of faces tilted 90° to either side of vertical [52].

The finding that adaptation still occurs with inverted images does not rule out a face-specific site for the adaptation (for inverted faces do strongly activate the FFA) [53,54]. Moreover, recent studies have pointed to more subtle orientation-dependent asymmetries in the adaptation effects. For example, after-effects for perceived identity may be stronger in upright faces [55], and adaptation to upright faces may show stronger transfer to upside-down faces [17,47] or contrast-negated faces [42] than vice versa. Moreover, after-effects from inverted faces (e.g. with varying eye height) may show greater transfer to non-face shapes (e.g. to an inverted ‘T’), suggesting that the after-effects for upright images may be more selective for faces [56].

### (c) Same versus different objects

A further approach to testing the level of processing revealed by the adaptation has involved testing how adaptation transfers across different classes of objects. For example, after-effects for distortion [12], gender [23] or expression [23,57–59] are strongest when the adapt and test images are drawn from the same individual, but both effects also show substantial transfer across different identities. Thus, adaptation can adjust to whatever the abstracted configural properties are that define facial attributes (though this in itself does not require a sensitivity change at the level at which these attributes are explicitly represented).

The extent to which the adaptation to a face is specifically selective for faces, and how it depends on the visual versus conceptual attributes of the stimuli, remains unclear, for the results thus far present a complex picture. Examples of the types of questions aimed at identifying the levels of processing underlying face adaptation including the following. (i) Does the adaptation depend on local features or global configuration? Adapting to isolated features (e.g. curved lines) can induce changes in the perceived expression of a face [60]. However, hybrid faces formed by combining features from opposing expressions do not produce adaptation [61]. Moreover, consistent features but in scrambled configurations, or non-face features shown in consistent configurations, both produce expression after-effects, suggesting that the adaptation was directly to the expression information whether this was carried by the local or global features [61]. (ii) Is the adaptation adjusting to a visual image or an abstract concept? Expression after-effects transfer across different images of the same person, but are not induced by non-facial images of emotion (e.g. an aggressive dog) or emotional words [59]. Adaptation to gender also does not transfer between images of faces or hands, though each category shows an after-effect [62], and in fact attractiveness after-effects can be induced for either faces or bodies [63]. However, a recent study found that adapting to male or female faceless bodies did induce a gender after-effect measured in faces [28]. Moreover, identity after-effects have also been reported following exposure to an individual's name [64], or after imagining an individual [65], and thus could potentially be mediated by visualization. Yet, a recent report found that real and imagined faces induced after-effects of opposite sign [66]. (iii) Does the adaptation depend on physical similarity or perceived similarity? The stimulus variations defining face images can be varied to produce equivalent physical changes that are categorically perceived to belong to either the same or different identities. Behavioural after-effects depend on whether the adapt and test images fall within or between these perceptual categories [67] (while functional magnetic resonance imaging (fMRI) responses to faces have found evidence for coding dependent on both physical similarity or perceived similarity [68,69]). The importance of perceived similarity is consistent with the finding that varying the low-level properties of the images (e.g. colour or spatial frequency content) leads to stronger contingent after-effects when these variations alter perceived identity [42]. (iv) Does the adaptation require awareness? Tilt or colour-contingent after-effects, or orientation-selective losses in contrast sensitivity, can be induced even when the adapting stimuli are too rapidly modulated in time or space to be perceptually resolved, and hence fail to be consciously detected [70,71]. After-effects for face identity are instead strongly suppressed by binocular rivalry, pointing to a later site for the adaptation [72]. Notably, the perception of threat-related expressions is less dependent on awareness [73,74]; yet, adaptation to expressions may also require attention [75].

### (d) Neural correlates

Neural measures of face adaptation have also been used to examine the nature and potential sites of response changes. For example, prior adaptation reduces the magnitude of the N170 component of the event related potential [62,76–78] and the corresponding M170 response in magnetoencephalography (MEG) [79] signals associated with an initial stage of detection of a stimulus as a face. The adaptation is category specific but can show strong transfer across different renditions of faces or even from isolated face parts [80]. Adaptation of the MEG response has also been used to implicate a role in expression adaptation of the superior temporal sulcus, an area associated with changeable characteristics of faces [81].

The blood oxygen level dependent response in fMRI also shows rapid adaptation to a repeated stimulus [82,83] and has been used in several studies to probe the properties of face adaptation [68,69,84–89]. Notably, the adaptation effects are widespread in the cortex, including regions beyond areas like the FFA that are identified through standard face localizer scans [86,90]; if these areas exhibit strong selectivity for individual facial characteristics they would not be strongly activated by face versus non-face comparisons [86]. Intriguingly, two recent studies have also reported that individuals with developmental prosopagnosia who thus have impaired face recognition, nevertheless exhibit largely normal face after-effects [91,92].

The diversity of these results concerning the nature of face adaptation is not surprising given that face stimuli will engage and potentially adapt many levels of visual processing, and that face perception itself may depend on a number of distinct processes with different characteristics for representing attributes such as identity and expression. However, together they strongly suggest that adaptation to faces depends, in part, on response changes at high and abstract levels of visual coding.

## 6. Selectivity of face adaptation for facial attributes

In the preceding section, we reviewed results implying that at least some part of the adaptation to faces taps directly into the visual representation of faces. If one grants this possibility (and even if one does not), then it becomes of considerable interest to ask how adaptations to different facial characteristics interact. Many of the studies addressing this question have asked whether adaptation to one facial attribute (e.g. gender) is contingent on the value of a different attribute (e.g. ethnicity), in order to assess whether different attributes are represented independently (in which case adaptation should transfer) or conjointly (in which case it should not). A number of dimensions exhibit partially selective and thus dissociable after-effects, including gender, age, ethnicity and species [86,93–98]. For example, opposing distortion or gender after-effects can be simultaneously induced in Asian versus Caucasian faces by adapting to a sequence of, say, Asian male and Caucasian female faces [86,95]. However, in other cases there is almost complete transfer. An intriguing example is that identity after-effects show little dependence on the expression of the adapting face [99], even though as noted above differences in identity reduce adaptation to expressions [23,57–59]. One possible explanation for this asymmetry is that different expressions may be encoded as variations on the same ‘object’ and thus may behave equivalently in identity adaptation, whereas a change in identity may cause the adapt and test to appear to be drawn from different ‘objects’ and thus lead to less transfer of the adaptation [42].

Several studies have also examined how face adaptation depends on differences in viewpoint (e.g. frontal versus three-quarter view images). These have found that after-effects show substantial transfer but are nevertheless strongest when the adapt and test images share the same perspective [19,25,38,39,64, 100–104]. One implication of this result is that facial identity may be encoded by multiple mechanisms selective for different viewpoints rather than abstracted out so that it is independent of the observer's perspective. Consistent with this, adapting to a face seen from a particular angle biases the perceived viewing angle of faces seen subsequently [105]. Like the after-effects for facial configuration, these viewpoint after-effects may also involve adaptation at high levels of visual coding. For example, the after-effects for different head orientations can be strongly biased by the physically minor but visually salient changes in the direction of gaze [106].

These results show that many of the dimensions along which faces appear to vary are not represented completely independently—at least at the levels affected by the adaptation. This is perhaps surprising given that we can usually readily judge one attribute (e.g. age or gender) regardless of others. However, there are clear parallels to these effects in the visual coding of simpler properties like colour and form. For example, adaptation to colour can be strongly selective for form [107,108], and thus reflects mechanisms that are selective for conjunctions of these features, even though these attributes behave as separable dimensions that can be accessed independently in tasks such as visual search [109]. A further important parallel is that this joint selectivity means that how adaptation modifies visual coding for one dimension can depend on the levels of other dimensions. This could suggest that norms may be established independently for different face categories, e.g. for male or female faces and different ethnicities, so that an Asian male/Caucasian female adapting sequence would shift the Asian norm male-ward and the Caucasian norm female-ward. We consider models for these effects further below, but here note that while separate norms may exist as prototypes of different types of face, the contingent after-effects do not show that those individual category norms have a role to play in the process of adaptation. Instead, these effects can be accommodated by mechanisms that all represent trajectories from a common prototype or general face norm.

## 7. Adaptation as renormalization

One of the most important principles to emerge from studies of face adaptation is that the after-effects are consistent with a norm-based code, in which individual faces are represented by how they deviate from an average or prototype face. That is, by this account the average face is special, because all other faces are judged relative to it. Norm-based accounts are central to many models of face space [9]. Among the most compelling evidence for prototype-referenced coding are facial caricatures, which are created by exaggerating the specific ways that an individual face differs from the average [110–112]. This suggests that faces might be represented by their polar coordinates in a multi-dimensional space where the angle defines their individual character and the vector length the strength of that individual character. Consistent with this account, single cell and fMRI responses of face-selective neurons suggest that the cells are tuned to encode the distinctiveness of individual faces from the average face [87,113]. This coding scheme contrasts with exemplar or central tendency models where each face is instead encoded by matching to a set of candidates—for example, according to the most active channel, among a highly selective set, for a given face [114,115]. In this case, there might be multiple channels spanning a given face dimension, with multiple cross points (figure 5), and thus there may be no level or neutral point that is special. Multiple-channel codes of this type are found for some low-level stimulus dimensions such as spatial frequency [116] and may also underlie some aspects of face perception including the direction of gaze [117,118]. However, many perceptual dimensions appear to be represented relative to a norm, which itself appears neutral and which thus reflects a qualitatively unique state [119]. For example, many simple shape attributes like aspect ratio or orientation show evidence for a special norm (e.g. circular or vertical), and the phenomenology of colours is organized analogously to the norm-based account of face space, by describing the direction (hue) and degree (saturation) that the colour differs from a norm (grey). Thus, in vision norms may be the norm.

Three properties of face after-effects have been used to implicate the presence of a special norm. All are based on the evidence that adapting to a new face renormalizes the face space so that the perceptually neutral norm, which by analogy with colour we may regard as a ‘neutral point’ in face space, is shifted towards the adapting face.

**Figure 3.**
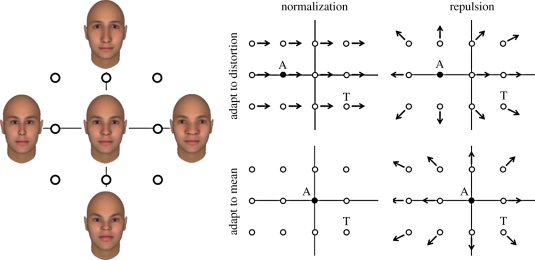
After-effects induced at different points in face space predicted by renormalization (left plots) or repulsion (right plots). Uniform renormalization predicts a constant shift in the appearance of all faces after adapting to a non-average face (top left), while no after-effects after adapting to the norm (bottom left). Repulsion predicts no shift in the appearance of the adapting image, and shifts away from the adapt for surrounding faces, with similar after-effects predicted for both non-average (top right) and average (bottom right) faces.

The first piece of supporting evidence is the asymmetry of the after-effects for neutral versus non-neutral faces. Webster & MacLin [12] found that adaptation to distorted faces strongly biased the appearance of a neutral (undistorted) face. Conversely, adapting to the neutral face did not alter the appearance of a distorted face. This is consistent with renormalization because the neutral face simply reinforces the current norm or neutral point in face space and hence changes nothing, whereas the distorted adapting face induces a shift in the neutral point so that the previously neutral faces are no longer seen as such (figure 3). Analogously, in colour vision, the spectral sensitivity is changed by introducing a long- or short-wavelength adapting light, but not by a light for which the relevant cone inputs are in the typical balanced state [120].

The second property is that adaptation changes the appearance of the adapting face itself so that it looks less distorted or distinctive, and produces appearance shifts in the same direction for faces that are either more or less distinctive than the adaptor [14,121]. (For example, adapting to a distended face causes all faces including the adapting face to appear narrower.) This suggests that the adaptation is to a first approximation simply recentring the perceptual space nearer to the current adapting stimulus (e.g. so that the physically distended adapting face now appears more neutral, or closer to the norm). Importantly, as discussed below, both of these properties contrast with the predictions of a template or exemplar-based model, where the visual system instead becomes less sensitive to the adapting face and to its near-neighbours in face space (figure 3). In this case, no shift is predicted in the appearance of the adapting face, and all other faces are predicted to appear less like the adapting face. Thus in this case, after adapting to a moderately distorted face, an extremely distorted face should appear even more distorted. Models of face adaptation that appeal only to low-level desensitization to the local contrast make the same false prediction, for similar reasons.

**Figure 4.**
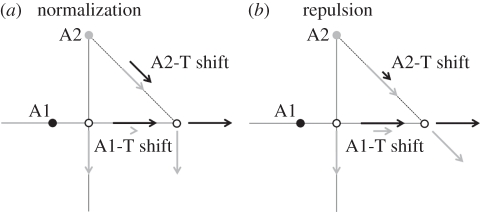
After-effects along trajectories passing either through (A1-T) or not through (A2-T) the norm, predicted by normalization or repulsion. (*a*) Normalization predicts each adapting face shifts appearance relative to the average. This produces the largest shifts along trajectories through the norm (A1-T) and weaker shifts along axes that do not intersect the norm. Along each axis the predicted shift (solid arrows) is given by the projection of the adapt vector (dashed arrows) onto the test vector, and thus by the cosine of the angle between the adapt and the test vectors. (*b*) Repulsion instead predicts that each adapt image will bias the test away from the adapt and thus predicts equivalent after-effects along either adapt–test axis for images at equivalent distances.

A third test for a norm in the adaptation process has involved comparing the after-effects for face dimensions that vary through the neutral point (face and antiface pairs) or along other directions in the space [16,122,123]. At the neutral point on this distortion continuum, the character perceptually attributed to the face switches from anti-face to face. As noted above, adapting to an antiface shifts this transition point so that the quality of the original face now appears in faces that would ordinarily have been perceived as belonging to the anti-face class. But when the same procedure is applied using a continuum of adapt and test distortions that does not pass near the norm, the after-effects appear substantially weaker. This again suggests that adaptation is specifically renormalizing the perception relative to a special neutral point. However, note that this does not mean that the adaptation interactions are specific to a particular face and its antiface. The average global shifts implied by simple renormalization instead predict that adapting to any face will shift the appearance of all faces along the identity axis in the direction that separates the norm from the adapting face [124]. Faces that are separated from the norm in different directions may undergo the same shift (e.g. after-effects to horizontal distortions are roughly the same regardless of the vertical distortions in the faces [12]); but if the after-effect is measured as the change in response along the adapt–test axis, then it will vary as the cosine of the angle formed by the identity axis and adapt–test axis (figure 4). Thus in the limit, no after-effect would be measured if the adapt to test axis was perpendicular to the identity axis. (By analogy, adapting to red will cause all colours to appear greener, but this after-effect would be less evident if the adapt and test colours are varied only along a red–blue axis.) A similar issue—that the axis of the after-effect may differ from the axis used to measure it—applies to interpreting measurements of the selectivity of the adaptation for different face attributes. It thus remains an important and unresolved question to ask in what ways adaptation to a given vector in face space alters the appearance of other directions, and the extent to which any changes reflect a global or more localized renormalization of the space.

## 8. Norms and the mechanisms of face coding

Models of visual coding typically assume that perceptual norms reflect a norm or neutral point in the underlying neural code [119]. That is, the norm looks special because the neural response is special. How could this norm be manifest in visual coding?

### (a) Norms along one dimension

**Figure 5.**
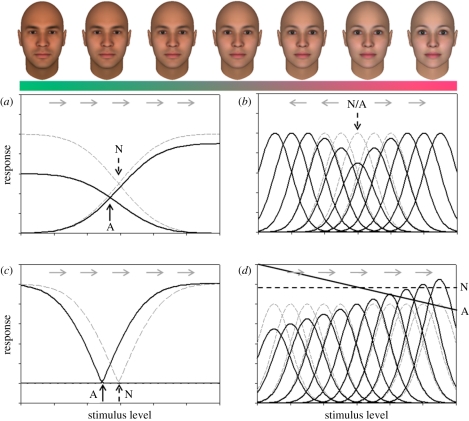
Norms and channel coding along a single perceptual dimension. The dimension may be represented by a small number of broadly tuned channels (left) or a large number of narrowly tuned channels (right). (*a*) The norm is represented implicitly by equal responses in the two channels. Adaptation to a biased stimulus reduces the response in one channel more than the other and shifts the norm from the one prevailing under neutral adaptation (N) towards the adapting level (A). This produces a shift in the appearance of all faces in the direction indicated by the arrows. (*c*) A split-range code in which the norm is represented explicitly by a null point between the responses of two channels that each respond to signals higher or lower than the norm (e.g. because each is formed by the half-wave rectified differences between the input channels at top left). Adapting to a new stimulus level (A) shifts the inputs and thus the null point. (*b*) If both the stimulus and the channels are narrowband, then adaptation will reduce the response at the adapting level and skew the responses of other stimuli away from the adapting level. In this case there is not a unique norm. (*d*) However, if the stimulus is broadband, then a norm is again implicitly represented by equal responses across the set of channels, and again renormalizes from the neutral stimulus (N) when the adapting stimulus is biased (A).

Figure 5 shows four different examples of how stimuli along a single dimension might be represented by a set of channels. As illustrated, the dimension might correspond to wavelength or hue (e.g. green to red), or to a facial characteristic (e.g. male to female). The response normalization suggested by adaptation has been explained by a model in which facial dimensions are encoded by a pair of mechanisms tuned to opposite poles of the dimension [8,121] (figure 5*a*). Similar ‘two-channel’ models have been used to account for opposing after-effects for orientation or shape [125,126]. In this case, the dimension is represented by the relative responses across the two channels, while the norm occurs at the unique point where the two responses are balanced. Adaptation is assumed to reduce each channel's response roughly in proportion to the channel's sensitivity to the adapting stimulus, and thus shifts the norm—and the appearance of all other faces—towards the adapting stimulus. This model is similar to colour vision at the level of the photoreceptors—where spectra are represented by the responses in a small number (three) of broadly tuned channels. At this level, the norm for colour vision can be thought to correspond to equal levels of excitation across the three cone classes [119]. This coding scheme can be contrasted with a multiple channel model in which the dimension is instead encoded by the distribution of responses across many channels that are each tuned to a narrow range of stimuli (figure 5*b*). In this case, adaptation depresses responses most in the channels tuned to the adapting level. This would not alter the perceived level of the adapting face (as the mode of the distribution does not change) while all other faces will appear shifted away from the adapting level. As noted, this repulsion is inconsistent with the observed characteristics of face adaptation.

However, there are alternative models that could also generate the same pattern of normalization. In the two cases above, we considered that the stimulus was narrowband in relation to the stimulus continuum of interest, while the channels were broad or narrow. Figure 5*d* illustrates the case where the channels are narrow but the stimulus is broad. Here, variations in the stimulus may correspond to changes in the slope rather than the peak of the activation profile across the channels, and a unique norm again occurs when the responses are equal across the bank of channels. Adapting to a biased slope will once more reduce responses most in the channels that are most stimulated, rebalancing the responses and thus shifting the norm in ways that will be functionally similar to the effects of narrowband stimuli in the two-channel model. Broadband stimuli may be more typical of natural stimuli. For example, most colours we see reflect subtle biases throughout the visible spectrum rather than a single wavelength, and the norm of white typically corresponds to a flat or equal energy spectrum. A similar example is the perception of image blur. Multiple channel models were developed, in part, to account for spatial frequency adaptation, which suggested that the visual system has multiple mechanisms tuned to different spatial scales. Adapting to a single frequency produces repulsive after-effects [127] and there is no ‘neutral’ frequency that is special—characteristics of the model in figure 5*b*. However, natural images contain a broad frequency spectrum, and for these there is a unique balance to the spectrum that appears perceptually of normal sharpness as if properly focused [128] (though this is not always tied to a single simple property of the amplitude spectrum like its global slope). Biasing the slope of the spectrum by blurring or sharpening the image disrupts this balance, and leads to a renormalization of perceptual focus [129]. Thus, whether or not there is a unique norm and how that norm is biased by adaptation depend on the properties of both the channels (narrow or broad) and the stimulus (narrow or broad) [119]. As a result, it remains possible that there are dimensions of facial variation for which the underlying channels might instead appear narrowly tuned, but for which the ‘spectrum’ defining the individual face is broad.

Figure 5*c* shows at lower left yet another way that norms might be represented, this time in terms of what MacLeod & von der Twer [130] call a ‘*split range*’ code, where the input continuum is divided at a physiological null point (the face norm), with separate rectifying neurons responding to inputs on opposite sides of that null point. This channel could be formed by taking the difference of the two individual sensitivity regulated responses in figure 5*a*, followed by rectification, or the portrayed behaviour could be approximated if mutual inhibition between the opposite signals drives the response of both towards zero near the null point [131]. This winner-take-all organization represents the norm explicitly as a null or weak response within the channel, rather than implicitly by the balance of responses across channels. This opponent organization is a hallmark of colour coding, and may be a functional requirement for any perceptual domain defined by norms. It also offers a substantial gain in efficiency over the single-sigmoid encoding at upper left, as each channel can devote its full response range to only half the stimulus range, resulting in a square root of two improvement in signal to noise over two independently noisy channels that both span the entire range [130]. We discuss the functional consequences of these encoding schemes in §9.

Face adaptation is often suggested to imply some form of an opponent code [8,21,117,132], but the effects thus far have not revealed an explicit opponent mechanism of the kind found for colour. In particular, it should be noted that negative after-effects do not require an opponent mechanism, and thus are not evidence for one. For example, a common mistake in the discussions of colour vision is to attribute complementary colour afterimages to opponent processing. The sensitivity changes underlying simple chromatic adaptation are instead largely receptoral—at the non-opponent stage of colour coding [133–135], and as Helmholtz [136] understood, the roughly complementary colour of negative afterimages is easily explained on that basis. The opposite after-effect that is induced results primarily from the rebalancing of responses across receptors that are adapting independently, and while the afterimage may appear phenomenally opponent, this does not necessitate an actual opponent mechanism (in the same way that the negative after-effect in figure 1 does not require an opponent mechanism for faces with positive and negative contrast). Psychophysical evidence for opponency instead required demonstrating that the signals from different cone types interact antagonistically to affect sensitivity [137,138]. Comparable parallels to this ‘second-site’ adaptation in face perception remain largely unexplored. One form of adaptation that directly demonstrates opponency is contrast adaptation [139,140]. Viewing a light that alternates between red and green or bright and dark reduces the perceived contrast of test lights, without changing the appearance of the mean (zero contrast). Opponency is demonstrated by the fact that the changes in sensitivity can be selective for colour or luminance contrast, whereas a single receptor would be modulated by both types of variation [139]. Tests of ‘contrast’ adaptation for faces—by exposing observers to a distribution of faces that vary around the average face—have found only weak hints of changes [141,142]. (Specifically, after adapting to faces that vary from markedly expanded to markedly contracted there is little if any reduction in the perceived extent of the distortions.) However, contingent after-effects, of the kind we discuss next, are consistent with a form of contrast adaptation in face space.

### (b) Norms along two or more dimensions

**Figure 6.**
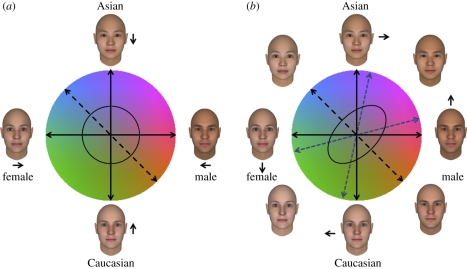
Norms and channel coding along two perceptual dimensions (gender and ethnicity). (*a*) The two dimensions represented by two independent channels. Adapting to stimuli that covary in gender and ethnicity (e.g. female Asian versus male Caucasian) should produce independent response changes along each axis and thus will not lead to contingent after-effects. (*b*) If the stimulus dimensions are instead encoded by multiple mechanisms selective for different combinations of the two attributes, then adapting to covarying stimuli will tilt the appearance of intermediate axes away from the adapting axis, resulting in contingent after-effects.

The models we considered so far are for encoding a single dimension, but can be extended to represent multiple dimensions. Figure 6*a* illustrates a representation of faces in terms of two dimensions—gender and ethnicity. We have overlaid this face space onto colour space to again emphasize the close parallels between this representation and models of colour coding. Again in colour vision, hue (identity) corresponds to the vector direction while saturation (identity strength) corresponds to the vector length relative to grey (the norm). Similarly, antifaces are analogous to complementary colours while ‘caricatured’ colours are hues that are over-saturated.

Only two axes are required to represent all the stimulus levels in the plane. However, suppose the two dimensions are instead spanned by multiple mechanisms, each activated by a different weighted combination of signals along the axes and therefore responding preferentially to stimuli that deviate from the norm in a particular direction in the plane (figure 6*b*) [86]. Now an individual hue or identity trajectory might be represented by the most responsive mechanism of this set. (Analogously, orientation is represented by many neural channels each tuned to a different preferred orientation [6].) Notably, this multiple-channel model behaves like a hybrid between a norm-based code (for saturation or identity strength) and an exemplar code (for hue or identity trajectory). In particular, with a uniform distribution of preferred directions, no direction in the plane is special, and each direction might be represented by the centroid of the distribution of activity across the channels.

How could this model be tested with adaptation? Webster & Mollon [140,143] examined changes in colour appearance after adapting to fields that varied along different axes and thus between complementary pairs of colours. If these stimuli were represented in terms of the independent signals along the two principal axes, then adaptation should always lead to independent response changes along either axis. This could reduce responses along each axis but should always lead to a selective compression of colour space along one of the primary axes (whichever is adapted more) or to a non-selective loss (if axes are adapted equally; figure 6*a*). Instead, they found that the adaptation was always selective for the adapting axis, an after-effect consistent with multiple selective channels each tuned to a different direction in the plane (figure 6*b*). The selectivity was revealed both in a greater loss of perceived contrast along the adapting axis, and in biases in apparent hue away from the adapting axis. For example, after adapting to the blue–yellow axis (along the negative diagonal), blues and yellows appeared less saturated, and all other hues appeared biased or rotated away from the adapting axis. No shifts were observed in the hue of the adapting axis. One interpretation of these results is that while simple chromatic adaptation (to a single colour) produces an adaptive shift in colour space, chromatic contrast adaptation (to a single colour axis) renormalizes the contrast [144] selectively along the adapting axis and others near it. On this view, colour contrast adaptation and simple chromatic adaptation have quite different mechanisms and different perceptual consequences. Simple chromatic adaptation induces a relatively uniform shift in colour space, while colour contrast adaptation gives rise to a perceptual repulsion from the adapting hue axis.

Analogous tests could be applied to face space, and in fact have been. Instead of adapting to an alternation of complementary colours, the observer adapts to opposed pairings of facial characteristics, in a sequence balanced around the norm along a diagonal axis (e.g. Asian female face versus Caucasian male face) (figure 6) [42,67,86,94–98]. Contingent after-effects typically result (e.g. a male-ward shift in the perception of Asian faces, together with a female shift for Caucasian ones). This shows that the two dimensions (gender and ethnicity) are not represented independently, and that the after-effect is not just a simple translation of phenomenal face space relative to physical face space. One way to amend that scheme is to suppose that adaptation induces not only the previously discussed perceptual shift towards the general face norm (which will ideally cancel out under adaptation to symmetrically paired faces), but in addition, a perceptual repulsion from the adapting face axis, analogous to the hue repulsion of Webster & Mollon [140]. For test stimuli on opposite sides of the origin, the repulsion shifts are opposite in direction. This view can explain the contingent after-effects without abandoning the assumption that adaptation always involves a shift relative to the general face norm; whatever more specific norms might be postulated, they need have no role in face adaptation. As Webster & Mollon [140] noted, the hue shifts following adaptation to different axes may be a form of ‘tilt after-effect’ in colour space. Similarly, the contingent after-effects that have been observed for different facial dimensions may be a tilt after-effect within face space.

An alternative to this account of contingent adaptation is that separable pools of mechanisms encode how faces within different categories differ from each category-specific prototype (so that face space is actually a collection of many category-specific spaces that each respond and adapt relative to their local norm) [94–97]. This leads to qualitatively similar predictions for adaptation to conjunctions (e.g. to a female Asian face), but it is less clear how such models can accommodate the lack of an after-effect induced by the global norm (as that face is distinctive relative to each local norm and thus should induce a change in the local norms). A further consideration is that many of the contingent effects can be explained by assuming that adaptation does not produce a uniform shift in face space but rather a shift that decreases with increasing distance between the adapt and test stimuli. By that account, a female Asian face will produce a larger gender after-effect in Asian than in Caucasian faces simply because the Asian faces are more similar to each other. Finally, the presence of a contingent after-effect does not require that the mechanisms are intrinsically tuned to the dimensions defining the faces, as this tuning could potentially arise on the fly if the underlying dimensions are not adapting independently [145].

### (c) Contrast in face space

The parallels we have drawn between face coding and colour coding associate facial identity trajectories with hue, and identity strength with saturation or contrast. However, unlike colour, the ‘contrast’ of a face stimulus can be varied in two ways: as the magnitude of the facial dimension (e.g. how female or male the face appears) or as the physical contrast of the image (e.g. how faded or distinct the image is). Both are potentially relevant for understanding the adaptation effects because both can modulate the neural response, and moreover both may be important for encoding some facial dimensions including gender [146]. Higher visual areas that encode objects and faces are less affected by variations in luminance contrast than cells in primary visual cortex [147,148], suggesting that identity strength is functionally the more important dimension of contrast. Conversely, an individual's face does not normally vary in its identity strength, raising the puzzle of why face identity is not encoded more discretely. One possibility is that categorical coding would greatly reduce the number of distinct identities that could be represented by the distribution of responses across the coding dimensions. A second puzzle is why, as we noted above, there is not a clear effect of adaptation to variations in identity strength comparable to the losses in perceived contrast that result from variations in colour saturation.

How the luminance contrast of a face affects, and is affected by, adaptation remains to be established. High-level shape after-effects show strong transfer across luminance contrast [149,150], consistent with the adaptation in higher visual areas. Yet, the limited results for face adaptation suggest that the distortion after-effect is much stronger for high contrast images [42]. Recent work has also shown that adaptation to faces selectively alters the contrast threshold required to recognize the face, suggesting that luminance contrast sensitivity may provide an additional metric for probing face after-effects [17,151]. At short durations, the adapting face actually facilitates luminance contrast detection of the same face and thus sensitivity losses are stronger for test faces that differ from the adapt face, suggesting that these effects may reflect a combination of both adaptation and priming.

### (d) The relationship between adaptation and norms in visual coding

As we noted at the outset of this section, this discussion hinges on the assumption that norms appear neutral because they lead to a neutral response in the actual neural code. An alternative is that norms are instead the learned characteristics of our environment and thus might be related to neural responses in arbitrary ways. For example, there are large individual differences in the stimulus that observers perceive as achromatic [152] (or, presumably, as an average face). This could be because observers are each normalized in a different way (perhaps because they are adapted to different colour environments), but could also be because they adopt different criteria for labelling a stimulus as white. In fact this distinction is central to the debate over cultural versus biological determinants of norms for colour attributes like basic colour terms [153] or for facial attributes like attractiveness [154]. Adaptation provides a means to test these alternatives [119,155]. If the norm corresponds to a balanced response across the channels, then adaptation to the norm should not alter the response, or in other words should not induce an after-effect. That is, the face stimulus that looks neutral should be the stimulus that does not change the state of adaptation, just as the spectral light that appears achromatic also leaves spectral sensitivity unaffected [156,157]. Two observers with different norms should thus each show no after-effect when adapted to their own norm, but a bias when adapted to the neutral stimulus chosen by the other. Conversely, if the observers differ in their criteria but not the visual code, then the neutral adapting stimulus will be the same for both, and need not correspond to the subjective neutral point for either. Finally, if individual differences in subjective norms result from differences in how neural responses are normalized, then adapting all observers to a common stimulus should reduce the inter-observer variation in subjective norms. Applications of these tests to both colour and faces suggest that individual differences in norms are tied to individual differences in sensitivity, and thus that the stimuli that appear neutral are in fact tied to a neutral response state at the level(s) of the visual system affected by the adaptation [119,155]. Moreover, exposing individuals to a common grey, or to an average face, results in much greater agreement in their chosen norm. Thus, the perception of a special norm for colour or faces may have an actual neural basis that is intimately linked to the observer's state of adaptation.

## 9. Visual coding, adaptation and natural image statistics

Another approach to understanding the properties of neural coding is to ask how information about the stimulus is adapted—over even evolutionary timescales—to be represented optimally. This theoretical approach has led to important insights into early visual coding and may similarly reveal the principles involved in high-level representations. An example of how these analyses have been applied to the specific case of face processing can be found in Bartlett [158]. Here, we consider how optimal coding schemes derived from the analyses of colour vision can be used to predict how faces might be processed.

### (a) How is neural coding shaped by the statistics of the environment: colour as a test case

To be useful, neural representations should distinguish well among the kinds of stimuli that are usually encountered. When natural stimuli are represented by stimulus coordinates such as a distortion parameter for faces in a face space, or redness in a colour space, an efficient neural code requires a good match between the limited stimulus range over which the neural responding is strongly graded on the one hand, and the distribution of natural stimuli on the other. It is inefficient to waste discriminative ability on extremes of the stimulus continuum that are seldom encountered; but it is also less than optimal to neglect unusual cases totally, by devoting all discriminative capacity to just the most typical stimuli (near-whites, or nearly average faces). These extreme design choices imply an optimum code intermediate between them: steep gradients of neural response should be in the region where natural stimuli are concentrated.

But how can discriminative capacity be allocated at will to different parts of the stimulus space? Some latitude is generally available, by choosing suitable nonlinear functions for the dependence of neural responses on the stimulus dimensions. If discrimination is limited by random fluctuations or quantization errors introduced at the output of the nonlinear transformation—an important proviso—then discrimination in any region of the stimulus space can be improved by increasing the gradient of the stimulus-response function there. This happens because the uncertainty in the output corresponds to a smaller difference at the input: the equivalent random variation in the input will be inversely proportional to the gradient of the stimulus-response function. But since the limited range of neural responses precludes a steep gradient everywhere, an increased gradient and improved discrimination at one point entails a sacrifice in discriminative ability at another point where the gradient has been reduced.

### (b) Advantages of opponency: crispening and range matching

In both colour space [159] and face space [160], the natural stimulus distribution is centrally peaked. Near-greys are relatively common, and the margins of cone excitation space are only very sparsely populated [159]. In the case of face space, the defining coordinates are not well specified, but most measure on face images are likely to show the generally expected centrally peaked distribution, even in cases where discrete variation, notably in gender, makes the distributions closer to a mixture of Gaussians than to a single one. This means that fine discrimination is less important in the margins of colour space than in the centre, near white, where natural colour stimuli are most densely packed. Much psychophysical evidence on colour discrimination has indeed indicated best discrimination near white (or more precisely, near the stimulus to which the visual system is adapted, see below) with a progressive deterioration as the saturation of the reference stimulus increases [161–163]. This superiority of discrimination for the naturally abundant near-neutral colours originates from a compressive nonlinearity in the neural code at the colour-opponent level, where it is the differences between cone excitations that are represented [163]. With opponent neurons that respond in a nonlinearly compressed way to the imbalance of photoreceptor inputs, the steep gradients of the neural responses for nearly neutral colours—where the inputs are nearly balanced and the opponent neurons are not strongly activated—provide good discrimination for these naturally frequent stimuli, at the expense of reduced discrimination at the margins of colour space where it is seldom needed. The requirement for finer discrimination near white thus yields one important rationale for colour opponent encoding: paradoxically, colour-opponent neurons are best equipped to register deviations from the neutral norm to which they are relatively unresponsive.

Similar considerations favour a norm-based code for faces, as an efficient response to the statistics of the natural stimuli. The parallel may indeed be more than metaphorical, as any measure derived from a face image can potentially be encoded as a deviation from an established norm (with the norm as a null stimulus), and any such representation may ultimately require an opponent code. All frontally viewed faces, considered in purely physical terms, have considerable similarity to a face representing the perceptual norm. Opponent processing—a balancing of opposites mediated by mutual inhibition—is therefore a natural way to generate neural signals that are minimal or zero for the norm face. Even so basic an achievement as evaluation of shape independently of size could involve an opponent interaction between orientation-selective neural indicators of size or spatial frequency. Aspect ratio—a key parameter in face perception, especially under reduced conditions such as great viewing distance—can indeed be encoded more precisely than would be expected from independent assessments of horizontal and vertical extent [164], just as the red/green dimension of colour can be judged with far more precision than expected on the basis of independent assessments of the photoreceptor excitations. The verbal description ‘long-faced’ assesses aspect ratio relative to the face norm. The same may well be true of its neural representation, just as in colour vision the balance between redness and greenness places the transition point near the peak density of natural colours [159].

The advantages of the opponent code are exploited to good effect in colour discrimination. Yet, as we discuss below, studies of face discrimination show at best a fairly modest optimum in discrimination in the neighbourhood of the average face, with some dimensions (e.g. eye height or mouth height) consistent with an essentially linear response [21]. One possible reason for this discrepancy is that nonlinear encodings have no influence on discrimination if the limiting noise is introduced prior to the nonlinearity. In the representation of colour, significant nonlinearity is present already at the retinal output, where fluctuations of impulse counts at the retinal ganglion cells are an important source of added noise. But a norm-based representation of faces must originate centrally, at a stage where nearly all sources of noise may have already come into play. We discuss face discrimination further in §12 on functional advantages of adaptation.

### (c) Range matching

The natural variation along the intensity axis in colour space is much greater than along the red/green chromatic axis. Intensity varies with a standard deviation of about a factor of 2 or 3 under fixed illumination, but the red/green variations in the natural environment originate from surprisingly subtle variations in the balance of red- and green-sensitive cone photoreceptor excitations—a standard deviation of only a few per cent [159]. The contrast gain for colour must be set higher if the world is to appear perceptually balanced in colour and lightness. This is not possible by nonlinear compression of the photoreceptor inputs themselves, but an opponent neural signal for red/green balance can represent the subtle input imbalances that support colour, while achromatic intensity is represented by a parallel system with a correspondingly higher threshold and wider operating range [159, 165,166]. Comparable considerations arise in face space, although here the coordinates of the stimuli are not as well defined. Physically subtle deviations from the average along some continua, for instance the upturn or downturn of the mouth, or deviations from symmetry [121] may be as significant as grosser differences in other dimensions, and this could provide an incentive for representing them by relatively independent nonlinear signals. Adaptation may be helpful in this process of multi-dimensional range matching: independent, roughly reciprocal sensitivity-regulating processes for different directions in face space would provide a relatively simple way to match the operating ranges of such signals to the dispersion of the input, much as suggested by Webster & Mollon [140] for colour contrast adaptation. But as noted in §8, contrast adaptation in face perception so far appears weak at best. Perhaps, the computational expense of direction-selective adaptation in the high-dimensional face space is prohibitive.

### (d) Adaptively roving null points and contrast coding

Along each dimension, only a limited range of colours can be differentiated with precision, a necessary consequence of the limited dynamic range of neural signals. But the region of fine discrimination in colour space is not centred on white or any other fixed colour. Rather, it shifts to remain centred on the adapting colour, if a non-white condition of adaptation is defined by (for instance) a common background within which the colours are separately displayed [167]. The enhanced discrimination for colours close to the adapting background, sometimes termed ‘crispening’, occurs over a quite restricted range, with pronounced loss of discriminative ability at or beyond about 10 times the threshold colour contrast in each direction [168], a range roughly appropriate to the dispersion of environmental stimuli [130]. Face analogues of this effect have been more difficult to demonstrate, but they include the ‘other race’ effect discussed below.

### (e) Optimal nonlinearity

Definite prescriptions can be derived for neural input–output functions to optimize discrimination. Mutual information between input and output is maximized by making the slope of the response function inversely proportional to the environmental probability density function (histogram equalization [169]). Squared error of estimation is minimized with a milder nonlinearity that allows better discrimination in the margins of the space [130], but even this optimizing principle predicts a marked superiority of discrimination for nearly average faces, contrary to the findings that we discuss in §12. Important category distinctions may call for special treatment. In colour vision, interest in distinguishing fruit from leaves [170] may have dictated a redward shift of the perceptual norm, making the mode of the distribution of natural colours perceptually yellowish and greenish [159]; in face space, gender discrimination may be a comparable case. The high-dimensional case of face space can be handled similarly in principle, by range matching followed by (for example) multi-dimensional histogram equalization, if a coordinate system (not necessarily linear) can be found in which the stimulus probability distribution satisfies independence [130].

## 10. Adaptation and the tuning functions of face coding

We have seen that face after-effects can help to reveal the types of channel coding that the visual system might use to represent faces. Can it be further used to decipher the specific properties of faces to which these channels are tuned? Adaptation has played a central role in characterizing the stimulus selectivities of the mechanisms of visual processing. For example, studies of chromatic and chromatic contrast adaptation helped to reveal the spectral sensitivities of channels at different stages of colour vision [171], while as noted above, multiple channel models of spatial vision were in part founded on measurements of the selectivity of the adaptation for properties like the orientation and spatial frequency of patterns [6]. The rough correspondence between the bandwidths for frequency, orientation or colour measured psychophysically and in single cortical cells [172] gives some credence to the notion of adaptation as the psychologist's electrode. Can the same techniques be applied to tease apart the mechanisms of face coding?

Several problems hamper this. First, it is again not certain to what extent the adaptation is directly altering sensitivity at face-specific levels of processing. An adaptation effect at earlier levels is unlikely to reveal the stimulus dimensions to which face channels are tuned. Second, the selectivity of the adaptation for different natural facial categories does not require that the mechanisms of face coding are directly tuned to these specific categories, for as long as the mechanisms can be differentially adapted by different categories they will result in selective after-effects. Finally, the interpretation of any adaptation effects is muddied even further by the possibility that adaptation can itself change the tuning of the mechanisms. For example, natural variations in face configuration are such that simply derived neural signals will not be statistically independent, and one proposed function of adaptation is to improve the economy and precision of the neural representation by revising the channels' tuning so that their responses become independent [145,173,174]. Such models can in theory account for contingent adaptation between two dimensions even if these are represented independently before adaptation, and they predict contingent after-effects for any face dimensions that are associated in the stimulus.

Because of these problems, it remains unclear whether there are special axes in the representation of faces—perhaps corresponding to prominent natural categories—or if the selectivity of the after-effects simply reflects the distances between the stimuli in face space. Colour vision again provides a telling example that the stimulus dimensions that appear phenomenally special are not necessarily the dimensions to which the neural mechanisms are tuned. Hering's opponent process theory was founded on the observations that certain colours appeared unique and mutually exclusive (red or green, blue or yellow, and light or dark) [175]. However, opponent neurons in the retina and geniculate are tuned for a pair of directions in colour space distinctly different from the Hering directions [176]. Moreover, at cortical levels both adaptation and single-unit recording reveal multiple mechanisms tuned to different colour-luminance directions, so that the neural basis for special hues becomes less certain [172]. One possibility is that at later stages, the visual system builds cells with tuning functions that match colour appearance—so that there are mechanisms that directly signal the sensations such as pure blue and yellow [177]. Yet another alternative is that blue and yellow only appear special because they are special properties of the environment (phases of daylight) and not because they reflect special states of neural activity [178]. This question remains actively debated in colour science [179,180]. The point is that we cannot be confident that facial attributes that appear salient or ecologically relevant are the stimulus attributes that face processing is directly encoding.

## 11. Adaptation and facial expressions

One case where it might currently be possible to apply selective adaptation to dissect the tuning functions of face-coding mechanisms is facial expressions. Expressions represent highly stereotyped variations in facial configurations that correspond to well-defined action patterns. These action patterns are to a large extent universal and innate [181], and thus it is possible that visual mechanisms could be specifically tuned to these stimulus dimensions. Moreover, just as there are primary colours, there are only a small set of six basic expressions (joy, anger, sadness, surprise, fear and disgust) [182]. Thus, compared with facial identity, the ‘space’ of expressions may more nearly approach the space of colour in having only a small number of dimensions that can be clearly defined and that have stronger *a priori* connection to the space of possible visual channels. Adaptation could thus be used as a test for these channels. In particular, tests of the selectivity of after-effects could be used to ask whether each expression can be adapted (and thus might be represented) independently, or whether expressions might instead be coded by their directions within a space defined by a different set of cardinal axes corresponding to dimensions such as positive or negative valence or arousal. Such norm-based spaces are prevalent for representing expressions and emotion, and imply that different expressions should be complementary [183]. Tests for complementary after-effects could therefore provide evidence for or against this organization.

Strong facial after-effects can be induced by exposure to facial expressions [10,23,24,59,184]. For example, Webster *et al*. [23] found that adaptation biased the perceived boundary between two morphed expressions, paralleling the after-effects they found for gender or ethnicity. Hsu & Young [24] instead morphed between a neutral and expressive face, and found that sensitivity to the expression decreased following adaptation. These after-effects appear to be driven primarily by the visual characteristics of the stimulus. For example, adaptation to emotion conveyed by non-facial images or auditory stimuli produces little after-effect in faces [59], while stimuli with appropriate configural or featural information for an expression can induce a change [60,61]. Thus, these studies suggest that the adaptation is altering the responses of visual mechanisms and not processes related to an amodal concept of different expressions or emotions.

**Figure 7.**
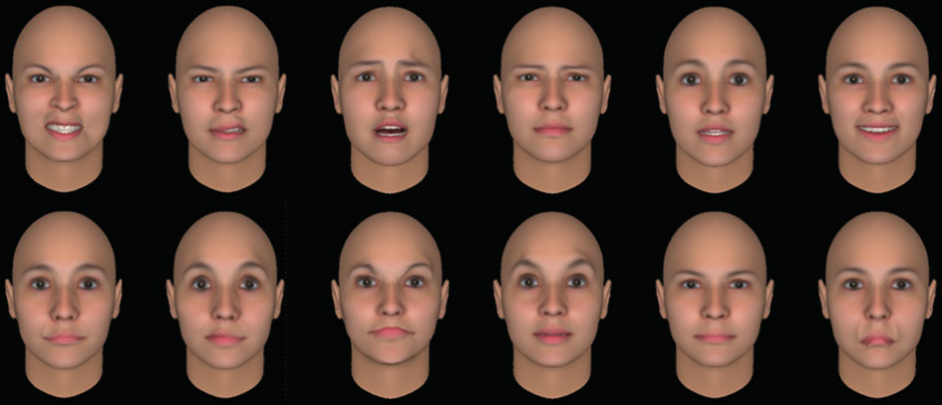
The six basic expressions (top row) and their anti-expressions (bottom row), corresponding to faces with the opposite configuration.

These after-effects are clearly strongest when the adapting and test faces have the same expression, yet it is less clear to what extent the different expressions adapt independently. For example, Hsu & Young [24] found that adapting to a sad face facilitated perceiving a face as happy and vice versa. Similarly, Rutherford *et al*. [184] observed that happy and sad faces produced complementary after-effects in a neutral face. In contrast, Juricevic & Webster [185] found little consistent change in sensitivity to each of the basic expressions after adapting to a different expression. (It should be noted that none of these studies measured changes in actual sensitivity, but rather measured which expression was reported for the face.) Complementary after-effects might be expected to occur for pairs of expressions that represent opposite spatial configurations. However, actual expressions may involve largely uncorrelated variations in facial structure [186]. That is, an ‘antihappy’ configuration does not clearly convey anger or sadness. Figure 7 illustrates this point by showing each of the six basic expressions and their anti-expressions formed by morphing through an average neutral face. The original expressions are largely identifiable, yet their visual opposites are surprisingly ambiguous.

Rather than an opponent organization, the most appropriate geometrical idealization of the phenomenal space for visually expressed emotions may instead consist of six monopolar and mutually roughly orthogonal dimensions radiating from the neutral norm. In a progression of morphs displaying decreasing intensities of a basic emotion, the appropriate way to continue the series past the neutral face may be obvious on physical analysis (the upturned or downturned mouth, for example), but there is generally no clearly corresponding phenomenal continuity in the anti-expressions. This echoes the phenomenology of identity, where antifaces tend to be viewed as unrelated individuals rather than specifically opposite, even though they are indeed opposite at the stimulus level [16]. And in this respect, hue is once again a parallel case, as we have argued above. In all three cases (expression, identity, hue), it is plausible that the monopolar nature of the phenomenal attributes is traceable to the rectifying behaviour of spiking neurons in the respective neural representations. But it may also have justification on functional grounds. The expression of each emotion is a distinct action, just as each hue results from a distinct mode of filtering of a broadband source. The case of emotional expression is unique, however, in that there is often no natural basis for the anti-stimulus to arise. For example, for expressions that include opening the mouth from the neutral face, there is no corresponding natural configuration for further closing the mouth.

This lack of symmetry in the frequency of occurrence between expressions and their opposite configurations raises the question of how the visual coding of facial expressions is normalized. If happy and angry faces were configural opposites, then adaptation could readily adjust the neutral point to the average between them. But if the anti-expressions instead correspond to expressions we are unlikely to experience, then it is no longer apparent what the counterweight is that holds the neutral face in place—if the adaptation depends on mechanisms that are directly representing the expressions. Perhaps instead, the normalization is set at a level of basic configural coding, shared with mechanisms involved in recognizing individuals [187]. This possibility is also consistent with evidence that adaptation to expressions is weaker when the identity of the test and adapt faces differ [57–59] as expected, if the adaptation occurs at sites before separate expression and identity representations are extracted. Surprisingly however, as we noted above, the converse selectivity is not found—identity after-effects are unaffected by a change in expression [99]. Another possible account of expression normalization is that once a general face norm is created, the deviations from that norm that express the six basic emotions are encoded by six monopolar signals, and the role of adaptation is just to adjust appropriately their sensitivity and operating range, always using the general face norm as the zero of their operating ranges.

## 12. Functional consequences of adaptation

What role do short-term adaptations of the kinds measured in face after-effects play in aiding perception? Most studies directed at this question have examined whether adaptation improves discrimination among faces. Some hint that this should occur is suggested by the ‘other race’ effect. It is generally easier to recognize or distinguish between individuals of one's own race (or rather, of the race one is exposed to). This is broadly analogous to the crispening effect in colour discrimination noted above, where it is easier to discriminate between colours that are both close to the white point or to an adapting background, than between colours that each contrast strongly with the adapting stimulus [168]. Discrimination is impaired for high-contrast stimuli because the retinal signals, which encode contrast, are saturated [159]. Similarly, in face perception it may be that we are better at discriminating among near-average faces, because the gradients of the neural signals are steepest around the average. Adaptation would then be important for centring the response and thus sensitivity around the current average, so that under experimentally contrived adaptation, the stimulus region where discrimination is best shifts along with the perceptual norm.

Tests of these ideas have met with mixed results. In Wilson *et al*. [188], discrimination thresholds for synthetic faces were lower around the average face than around atypical faces. Tanaka *et al*. [189] found that morphs physically half-way between a typical and an atypical face appeared subjectively more similar to the atypical face as expected if the centre of the face space is perceptually enlarged relative to the margins. Tanaka & Corneille [190] further found that near-average face morphs are discriminated with slightly greater precision than atypical ones. Dakin & Omigie [191] found a slight reduction in discrimination threshold for faces close to the average, but differing slightly from it. Rhodes *et al*. [192] found that differences among near-average faces are perceptually compressed rather than enhanced, but in some experiments found evidence of perceptual expansion near the middle of the range of experimentally presented faces. Finally, Susilo *et al*. [21] observed that discriminability of eye or mouth height was the same whether the feature positions were near or far from the norm. Some experimental studies of adaptation have found improvements in discrimination as adaptation progresses. Rhodes *et al*. [193] found that adapting to faces from a new ethnicity improves discrimination performance (identity thresholds) for faces drawn from that ethnicity. In other cases, the adaptation does not seem to help or hinder discrimination [19,192,194].

There are two potential reasons for the failure of adaptation to clearly impact distinguishing faces. First, the weak effect is not unique to faces but is found for most stimulus dimensions [195]. For example, adaptation to different contrast levels (as opposed to different mean light levels) has been extensively studied but has only rarely been found to improve contrast discrimination [171,196]; and while pattern discriminations show more promise, the improvements with adaptation are again not clearly robust [197–199]. For nonlinearity to influence discrimination strongly, the discrimination-limiting noise must be introduced after the nonlinearity (‘Birdsall's theorem’ [200]). This reduces the scope for characterizing central nonlinearities on the basis of discrimination data. Second, in a discrimination task, observers will use the best cues available, and thus it is difficult to ensure that their performance is limited by processes directly related to face perception. For example, the faces could in principle be distinguished by judging a simple ‘non-face’ feature like eye separation, in a representation designed for spatial distance judgements generally [192,201]; if so, the statistics of faces will be irrelevant. But as there is no alternative ‘non-colour’ basis for colour discrimination, performance in this case should depend entirely on the design of the neural code for colour, thereby revealing fully the influence of the natural distribution of environmental colours.

While adaptation thus seems to produce only weak changes in discrimination, it produces striking changes in appearance. Are there functional benefits to these appearance changes? One should not necessarily expect this, as face adaptation could be, in part, a side effect of luminance and contrast-adaptation processes that have been installed in the visual system for other purposes than face processing specifically. In colour vision, adaptation plays an important role by contributing to colour constancy [202]. The light spectrum reaching the eye from an object depends on both the reflectance function of the object and the spectrum of the illumination. In order for colour to reliably signal the object's reflectance, variations in illumination must therefore be discounted. Renormalizing the cone sensitivities so that the response to the average ambient spectrum is balanced tends to factor out changes in the general illumination and thus promotes constancy. It is conceivable that adaptation could play an analogous role in face perception [203]. In particular, constancy requires removing the response to irrelevant information from the stimulus. In faces, this could include the average characteristics of the population (e.g. their ethnic features). Normalizing to this average might thus remove uninformative features of the face (group characteristics) and allow informative cues to identity and expression (individual characteristics) to be perceived more directly. (While we cannot know the inner experience of another, an intriguing prediction of this adaptation is that different observers may experience the average face—just as the average colour—in phenomenally similar ways, yet for each, this average is tied to their specific population. That is, it is possible that the perceptual experience you have, looking at the average face to which you have been exposed, is fundamentally similar to the experience I have looking at mine [203].)

The processes of perceptual constancy are also important for removing uninformative variations in the observer. For example, spectral sensitivity can vary dramatically over time because of changes in the density of the lens pigment or across retinal location because of variations in macular pigment [119,204, 205]. A critical aspect of colour constancy is thus to compensate for the intrinsic sensitivity limitations of the observer. Renormalizing colour appearance for the average spectrum reaching the receptors can again partially factor out these sensitivity differences, and additional processes may contribute to even stronger constancy [206]. Such compensatory processes are likely to be important for calibrating or ‘error-correcting’ most perceptual dimensions so that the perception of patterns remains stable across factors such as changes in retinal location or viewpoint. Similarly, for face perception, an important function of adaptation may be to calibrate face-coding so that the population of mechanisms is normalized to consistent levels. Even though the adaptation to faces is very rapid, such adjustments may be particularly important for maintaining face coding over longer timescales involving large physiological changes, for example, during development or ageing. Indeed, it is possible that most of the functional consequences of adaptation involve initially calibrating the visual system, and that the after-effects that are observed experimentally are merely fine-tuning.

Finally, in the same way that constancy is important for calibrating different neural processes within the observer (so that they signal the same information despite different physiological limits), adaptation is also arguably important for equating perceptual responses across observers [203]. That is, adaptation may allow individuals with very different visual systems to perceive some aspects of the world in similar ways—by normalizing the idiosyncrasies of their coding to a common environment. A social consensus for facial characteristics (e.g. that average faces are more attractive) may depend on a perceptual consensus shaped by adaptation to a common visual world. An intriguing case where this socially shared norm may fail is in autism. Autistic individuals can show severe social impairments and deficits in face perception, and they have also been found to show weaker adaptation to faces, which could be a cause or effect of their problems in responding to faces [207].

## 13. Adaptation in the real world

As we have noted, the effects of adaptation are important not only for dissecting how faces are represented by the visual system, but also for understanding how our perception of faces is shaped by the facial characteristics with which we come in contact in our daily lives. We are all exposed to a different diet of faces, and if we are adapted to this diet then we might each encode and perceive faces in uniquely individual ways. For example, how old someone looks—in the literal sense of looking—may be very different for someone surrounded by a younger or older cohort. However, this presupposes that the adaptation measured in the laboratory is also manifest on the street. Is there evidence for this?

Thus far, the signs are mostly anecdotal (in that they cannot be tied with certainty to sensory adaptation), yet are nevertheless compelling. One example is that face adaptation effects are found when observers are engaged in natural visual tasks. An interesting case is portraiture. Asymmetries are difficult to detect while drawing a face but can become obvious when viewing the mirror-reversed image, a trick employed by artists. This effect could arise because the artist is adapting to and thus normalizing their sensitivity to the image they are drawing [35]. Further hints come from comparing facial judgements in observers who are known to have different exposure histories. For example, the effects of gender strength on ratings of facial attractiveness differ for students at same-sex versus mixed-sex schools in ways that are predictable from the average gender to which they are exposed [208]. Similarly, preferences for average faces differ cross-culturally in ways that are likely to reflect the relative exposure to different ethnic groups [209,210].

As noted above, a primary anecdotal source of evidence for face adaptation in natural viewing is the other-race effect, which could arise because we are best at distinguishing among faces that are near to the average face to which we are adapted. Another consequence is that we should become more sensitive to when a face differs from our own ethnicity. Japanese and Caucasian observers do show different average category boundaries in morphs between the two ethnicities, suggesting that for each group the characteristics defining the other group appear more salient [23]. If an observer moved to a new environment with a different ethnic mix, then we might expect their norm to shift accordingly. Webster *et al*. [23] tested this for Japanese students who were residents in the US. The ethnicity boundaries for long-term residents were significantly closer than new arrivals to the average category boundary for indigenous Caucasians, and the degree of the shift was correlated both with the length of time in the US and the degree of daily exposure to Caucasian faces. Judgements of attractiveness may also change as individuals join and thus adapt to a new population [210]—an observation noted by the anthropologist Malinowski [211].

Such results support the assumption that adaptation plays a significant role in calibrating face perception in natural viewing. And the effects of adaptation in the laboratory are so pronounced that it is difficult to imagine that it is not centrally involved in regulating our everyday experiences with faces.

## 14. Face coding and colour

We have drawn heavily on the domain of colour vision to illustrate how both long- and short-term adaptation processes modulate visual coding, in order to explore whether similar principles may play a role in face perception. The parallels between the norm-based coding schemes characterizing both colour and face processing suggest that the visual system may often reinvent common strategies to represent diverse characteristics of the visual world. Moreover, for both representations, adaptation may play analogous and central roles, suggesting that there are principles and properties of adaptation that are common throughout the visual stream.

Yet the functional role of face adaptation, and of the face space in which adaptation is modelled, remain tantalizingly obscure. The representation of faces in a metric space by associating neural signals with the various coordinates is a natural one and a useful tool for analysis, but it is not independently validated by introspection, or well-suited to subserve face recognition except indirectly. A metric representation of colour makes some functional sense, as in colour, continuous variations are significant: colour coordinates can map onto behaviourally significant distinctions, as with ripeness of fruits for instance, and achieving colour constancy under varying illumination calls for metric computations. With faces, there is a more limited role for continuous variation. Face recognition instead depends on a discrete yet holistic organization, somewhat like that of spoken words in a speaker's lexicon, and seems to proceed without any introspectively accessible reference to the face norm or to the coordinates of face space [212]. Indeed, colour space as well as face space are seriously incomplete as models of face and colour perception. If opponent signals were the basis of our recognition of colours, it would be easier to make discriminations on the individual signals that jointly represent colours than on their conjunctions. Yet, in fact, one cannot easily say which of a green surface and a red one is the more blue; and the same/different judgement for colour is so much easier than the dimensional comparison that it cannot be based on introspective access to the opponent codes. With faces, this problem is exacerbated because so many more dimensions are needed to identify an individual uniquely, whereas in colour, three suffice.

In closing, we illustrate how adaptation can be used to ask whether there are even closer connections between colour and face perception. Faces naturally vary not only in their shape and texture but also in their basic chromatic properties. For example, male faces are on average slightly redder than female faces and this difference is sufficient to aid gender classification when shape cues are highly impoverished [213]. Moreover, an individual's face can vary considerably in colour with changes in health or emotional state, and natural colour variations modulate judgements of facial attractiveness [214]. In fact, one posited role for primate trichromacy is to support judgements of skin tones to assess the mood and fitness of conspecifics [215,216]. Given that colour can provide a reliable cue to differences both between and within observers, we might expect the mechanisms mediating face recognition to use both shape and colour information. Yamashita *et al*. [42] explored this by testing whether face adaptation can be selective for specific combinations of colour and shape. These experiments used a variant of the McCollough effect, in which observers are adapted to complementary colours paired with gratings at different orientations [107]. In achromatic test bars, this leads to strong negative after-effects for colour that are contingent on orientation. However, when red and green are instead paired with oppositely distorted faces, there were neither visible colour after-effects, nor were shape after-effects contingent on the colour. Similar null effects were found when different colours were paired with different facial expressions, even when these colours were varied along chromatic axes consistent with different expressions [217]. One implication of this result is that there are in fact limits to visual plasticity—shapes and colours that are clearly distinguishable cannot be arbitrarily associated through adaptation. This is important because it suggests that the contingent adaptation effects that have been reported for faces are actually revealing something about the tuning properties of face mechanisms [23,67,203], and that it is by design that the visual system combines these dimensions in a joint representation. A second implication is that while both colour and shape may be useful for evaluating faces, they may be encoded largely independently. The neural machinery deciphering the spatial configurations of faces may share many tricks with colour vision, yet may be colour blind.

## References

[1] Russell R. Sinha P. Biederman I. Nederhouser M. 2006 Is pigmentation important for face recognition? Evidence from contrast negation. Perception 35, 749–759 10.1068/p5490 (doi:10.1068/p5490) 16836042

[2] McKone E. Kanwisher N. Duchaine B. C. 2007 Can generic expertise explain special processing for faces? Trends Cogn. Sci. 11, 8–15 10.1016/j.tics.2006.11.002 (doi:10.1016/j.tics.2006.11.002) 17129746

[3] Gibson J. J. Radner M. 1937 Adaptation, after-effect and contrast in the perception of tilted lines. I. Quantitative studies. J. Exp. Psych. 20, 453–467 10.1037/h0059826 (doi:10.1037/h0059826)

[4] Kohler W. Wallach H. 1944 Figural aftereffects: an investigation of visual processes. Proc. Am. Phil. Soc. 88, 269–357

[5] Webster M. A. 2003 Pattern selective adaptation in color and form perception. In The visual neurosciences (eds Chalupa L. M. Werner J. S.), vol. 2, pp. 936–947 Cambridge, MA: MIT Press

[6] Graham N. V. 1989 Visual pattern analyzers. Oxford, UK: Oxford University Press

[7] Clifford C. W. G. Rhodes G. 2005 Fitting the mind to the world: adaptation and aftereffects in high-level vision, *Advances in visual cognition series*, vol. 2 Oxford, UK: Oxford University Press

[8] Rhodes G. Robbins R. Jaquet E. McKone E. Jeffery L. Clifford C. W. G. 2005 Adaptation and face perception–how aftereffects implicate norm based coding of faces. In Fitting the mind to the world: adaptation and aftereffects in high-level vision (eds Clifford C. W. G. Rhodes G.), Advances in visual cognition series, vol. 2, pp. 213–240 Oxford, UK: Oxford University Press

[9] Valentine T. 1991 A unified account of the effects of distinctiveness, inversion, and race in face recognition. Q. J. Exp. Psychol. A 43, 161–204 1866456 10.1080/14640749108400966

[10] Russell J. A. Fehr B. 1987 Relativity in the perception of facial expressions. J. Exp. Psychol. Gen. 116, 223–237 10.1037/0096-3445.116.3.223 (doi:10.1037/0096-3445.116.3.223)

[11] O'Leary A. McMahon M. 1991 Adaptation to form distortion of a familiar shape. Percept. Psychophys. 49, 328–332 10.3758/BF03205988 (doi:10.3758/BF03205988) 2030929

[12] Webster M. A. MacLin O. H. 1999 Figural aftereffects in the perception of faces. Psychonom. Bull. Rev. 6, 647–653 10.3758/BF03212974 (doi:10.3758/BF03212974) 10682208

[13] MacLin O. H. Webster M. A. 2001 Influence of adaptation on the perception of distortions in natural images. J. Electron. Imag. 10, 100–109 10.1117/1.1330573 (doi:10.1117/1.1330573)

[14] Rhodes G. Jeffery L. Watson T. L. Clifford C. W. G. Nakayama K. 2003 Fitting the mind to the world: face adaptation and attractiveness aftereffects. Psychol. Sci. 14, 558–566 10.1046/j.0956-7976.2003.psci_1465.x (doi:10.1046/j.0956-7976.2003.psci_1465.x) 14629686

[15] Buckingham G. DeBruine L. M. Little A. C. Welling L. L. M. Conway C. A. Tiddeman B. P. Jones B. 2006 Visual adaptation to masculine and feminine faces influences generalized preferences and perceptions of trustworthiness. Evol. Hum. Behav. 27, 381–389 10.1016/j.evolhumbehav.2006.03.001 (doi:10.1016/j.evolhumbehav.2006.03.001)

[16] Leopold D. A. O'Toole A. J. Vetter T. Blanz V. 2001 Prototype-referenced shape encoding revealed by high-level aftereffects. Nat. Neurosci. 4, 89–94 10.1038/82947 (doi:10.1038/82947) 11135650

[17] Guo X. Y. M. Oruc I. Barton J. J. S. 2009 Cross-orientation transfer of adaptation for facial identity is asymmetric: a study using contrast-based recognition thresholds. Vis. Res. 49, 2254–2260 10.1016/j.visres.2009.06.012 (doi:10.1016/j.visres.2009.06.012) 19540870

[18] Hurlbert A. 2001 Trading faces. Nat. Neurosci. 4, 3–5 10.1038/82877 (doi:10.1038/82877) 11135632

[19] Anderson N. D. Wilson H. R. 2005 The nature of synthetic face adaptation. Vis. Res. 45, 1815–1828 10.1016/j.visres.2005.01.012 (doi:10.1016/j.visres.2005.01.012) 15797771

[20] Davidenko N. Witthoft N. Winawer J. 2008 Gender aftereffects in face silhouettes reveal face-specific mechanisms. Vis. Cogn. 16, 99–103

[21] Susilo T. McKone E. Edwards M. 2010 What shape are the neural response functions underlying opponent coding in face space? A psychophysical investigation. Vis. Res. 50, 300–314 10.1016/j.visres.2009.11.016 (doi:10.1016/j.visres.2009.11.016) 19944116

[22] Seyama J. Nagayama R. S. 2009 Probing the uncanny valley with the eye size aftereffect. Presence 18, 321–339 10.1162/pres.18.5.321 (doi:10.1162/pres.18.5.321)

[23] Webster M. A. Kaping D. Mizokami Y. Duhamel P. 2004 Adaptation to natural facial categories. Nature 428, 557–561 10.1038/nature02420 (doi:10.1038/nature02420) 15058304

[24] Hsu S. M. Young A. W. 2004 Adaptation effects in facial expression recognition. Vis. Cogn. 11, 871–899 10.1080/13506280444000030 (doi:10.1080/13506280444000030)

[25] Jiang F. Blanz V. O'Toole A. J. 2006 Probing the visual representation of faces with adaptation: a view from the other side of the mean. Psychol. Sci. 17, 493–500 10.1111/j.1467-9280.2006.01734.x (doi:10.1111/j.1467-9280.2006.01734.x) 16771799

[26] O'Neil S. F. Webster S. M. Webster M. A. In press. Adaptation and the perception of facial age. Vis. Cogn. 10.1080/13506285.2011.561262 PMC3247904 22215952

[27] Greenlee M. W. Georgeson M. A. Magnussen S. Harris J. P. 1991 The time course of adaptation to spatial contrast. Vis. Res. 31, 223–236 10.1016/0042-6989(91)90113-J (doi:10.1016/0042-6989(91)90113-J) 2017883

[28] Ghuman A. S. McDaniel J. R. Martin A. 2010 Face adaptation without a face. Curr. Biol. 20, 32–36 10.1016/j.cub.2009.10.077 (doi:10.1016/j.cub.2009.10.077) 20022246 PMC3023960

[29] Leopold D. A. Rhodes G. Muller K. M. Jeffery L. 2005 The dynamics of visual adaptation to faces. Proc. R. Soc. B 272, 897–904 10.1098/rspb.2004.3022 (doi:10.1098/rspb.2004.3022) PMC1564098 16024343

[30] Rhodes G. Jeffery L. Clifford C. W. Leopold D. A. 2007 The timecourse of higher-level face aftereffects. Vis. Res. 47, 2291–2296 10.1016/j.visres.2007.05.012 (doi:10.1016/j.visres.2007.05.012) 17619045

[31] Vul E. Krizay E. MacLeod D. I. 2008 The McCollough effect reflects permanent and transient adaptation in early visual cortex. J. Vis. 8(12), 4, 1–12 10.1167/8.12.4 (doi:10.1167/8.12.4) 18831617

[32] Suzuki S. Cavanagh P. 1998 A shape-contrast effect for briefly presented stimuli. J. Exp. Psychol. Human 24, 1315–1341 10.1037/0096-1523.24.5.1315 (doi:10.1037/0096-1523.24.5.1315) 9778826

[33] Kovacs G. Zimmer M. Harza I. Vidnyanszky Z. 2007 Adaptation duration affects the spatial selectivity of facial aftereffects. Vis. Res. 47, 3141–3149 10.1016/j.visres.2007.08.019 (doi:10.1016/j.visres.2007.08.019) 17935749

[34] Carbon C. C. Strobach T. Langton S. R. H. Harsanyi G. Leder H. Kovacs G. 2007 Adaptation effects of highly familiar faces: immediate and long lasting. Memory Cogn. 35, 1966–1976 10.3758/BF03192929 (doi:10.3758/BF03192929) 18265612

[35] Morikawa K. 2005 Adaptation to asymmetrically distorted faces and its lack of effect on mirror images. Vis. Res. 45, 3180–3188 10.1016/j.visres.2005.08.004 (doi:10.1016/j.visres.2005.08.004) 16169038

[36] McKone E. Edwards M. Robbins R. Anderson R. 2005 The stickiness of face adaptation aftereffects. J. Vis. 5, 822a 10.1167/5.8.822 (doi:10.1167/5.8.822)

[37] Carbon C. C. Leder H. 2006 Last but not least: the Mona Lisa effect: is ‘our' Lisa fame or fake? Perception 35, 411–414 10.1068/p5452 (doi:10.1068/p5452) 16619955

[38] Jiang F. Blanz V. O'Toole A. J. 2007 The role of familiarity in three-dimensional view-transferability of face identity adaptation. Vis. Res. 47, 525–531 10.1016/j.visres.2006.10.012 (doi:10.1016/j.visres.2006.10.012) 17207832

[39] Jiang F. Blanz V. O'Toole A. J. 2009 Three-dimensional information in face representations revealed by identity aftereffects. Psychol. Sci. 20, 318–325 10.1111/j.1467-9280.2009.02285.x (doi:10.1111/j.1467-9280.2009.02285.x) 19207696

[40] Kohn A. Movshon J. A. 2003 Neuronal adaptation to visual motion in area MT of the macaque. Neuron 39, 681–691 10.1016/S0896-6273(03)00438-0 (doi:10.1016/S0896-6273(03)00438-0) 12925281

[41] Kovacs G. Zimmer M. Harza I. Antal A. Vidnyanszky Z. 2005 Position-specificity of facial adaptation. Neuroreport 16, 1945–1949 10.1097/01.wnr.0000187635.76127.bc (doi:10.1097/01.wnr.0000187635.76127.bc) 16272884

[42] Yamashita J. A. Hardy J. L. De Valois K. K. Webster M. A. 2005 Stimulus selectivity of figural aftereffects for faces. J. Exp. Psychol. Human 31, 420–437 10.1037/0096-1523.31.3.420 (doi:10.1037/0096-1523.31.3.420) 15982123

[43] Zhao L. Chubb C. 2001 The size-tuning of the face-distortion after-effect. Vis. Res. 41, 2979–2994 10.1016/S0042-6989(01)00202-4 (doi:10.1016/S0042-6989(01)00202-4) 11704237

[44] Afraz A. Cavanagh P. 2009 The gender-specific face aftereffect is based in retinotopic not spatiotopic coordinates across several natural image transformations. J. Vis. 9, 11–17 10.1167/9.10.10 (doi:10.1167/9.10.10) PMC2792707 19810791

[45] Afraz S. R. Cavanagh P. 2008 Retinotopy of the face aftereffect. Vis. Res. 48, 42–54 10.1016/j.visres.2007.10.028 (doi:10.1016/j.visres.2007.10.028) 18078975 PMC2674370

[46] Kanwisher N. McDermott J. Chun M. M. 1997 The fusiform face area: a module in human extrastriate cortex specialized for face perception. J. Neurosci. 17, 4302–4311 9151747 10.1523/JNEUROSCI.17-11-04302.1997 PMC6573547

[47] Watson T. L. Clifford C. W. G. 2003 Pulling faces: an investigation of the face-distortion aftereffect. Perception 32, 1109–1116 10.1068/p5082 (doi:10.1068/p5082) 14651323

[48] Yin R. K. 1969 Looking at upside-down faces. J. Exp. Psychol. 81, 141 10.1037/h0027474 (doi:10.1037/h0027474)

[49] Freire A. Lee K. Symons L. A. 2000 The face-inversion effect as a deficit in the encoding of configural information: direct evidence. Perception 29, 159–170 10.1068/p3012 (doi:10.1068/p3012) 10820599

[50] Maurer D. Grand R. L. Mondloch C. J. 2002 The many faces of configural processing. Trends Cogn. Sci. 6, 255–260 10.1016/S1364-6613(02)01903-4 (doi:10.1016/S1364-6613(02)01903-4) 12039607

[51] Rhodes G. Jeffery L. Watson T. L. Jaquet E. Winkler C. Clifford C. W. G. 2004 Orientation-contingent face aftereffects and implications for face-coding mechanisms. Curr. Biol. 14, 2119–2123 10.1016/j.cub.2004.11.053 (doi:10.1016/j.cub.2004.11.053) 15589154

[52] Watson T. L. Clifford C. W. G. 2006 Orientation dependence of the orientation-contingent face aftereffect. Vis. Res. 46, 3422–3429 10.1016/j.visres.2006.03.026 (doi:10.1016/j.visres.2006.03.026) 16723149

[53] Kanwisher N. Tong F. Nakayama K. 1998 The effect of face inversion on the human fusiform face area. Cognition 68, B1–B11 10.1016/S0010-0277(98)00035-3 (doi:10.1016/S0010-0277(98)00035-3) 9775518

[54] Yovel G. Kanwisher N. 2005 The neural basis of the behavioral face-inversion effect. Curr. Biol. 15, 2256–2262 10.1016/j.cub.2005.10.072 (doi:10.1016/j.cub.2005.10.072) 16360687

[55] Rhodes G. Evangelista E. Jeffery L. 2009 Orientation-sensitivity of face identity aftereffects. Vis. Res. 49, 2379–2385 10.1016/j.visres.2009.07.010 (doi:10.1016/j.visres.2009.07.010) 19631682

[56] Susilo T. McKone E. Edwards M. 2009 Solving the upside-down puzzle: inverted face aftereffects derive from shape-generic rather than face-specific mechanisms. J. Vis. 9, 451 10.1167/9.8.451 (doi:10.1167/9.8.451) 21149314

[57] Campbell J. Burke D. 2009 Evidence that identity-dependent and identity-independent neural populations are recruited in the perception of five basic emotional facial expressions. Vis. Res. 49, 1532–1540 10.1016/j.visres.2009.03.009 (doi:10.1016/j.visres.2009.03.009) 19303422

[58] Ellamil M. Susskind J. M. Anderson A. K. 2008 Examinations of identity invariance in facial expression adaptation. Cogn. Affect. Behav. Neurosci. 8, 273–281 10.3758/CABN.8.3.273 (doi:10.3758/CABN.8.3.273) 18814464

[59] Fox C. J. Barton J. J. 2007 What is adapted in face adaptation? The neural representations of expression in the human visual system. Brain Res. 1127, 80–89 10.1016/j.brainres.2006.09.104 (doi:10.1016/j.brainres.2006.09.104) 17109830

[60] Xu H. Dayan P. Lipkin R. M. Qian N. 2008 Adaptation across the cortical hierarchy: low-level curve adaptation affects high-level facial-expression judgments. J. Neurosci. 28, 3374–3383 10.1523/JNEUROSCI.0182-08.2008 (doi:10.1523/JNEUROSCI.0182-08.2008) 18367604 PMC6670605

[61] Butler A. Oruc I. Fox C. J. Barton J. J. 2008 Factors contributing to the adaptation aftereffects of facial expression. Brain Res. 1191, 116–126 10.1016/j.brainres.2007.10.101 (doi:10.1016/j.brainres.2007.10.101) 18096142 PMC2538428

[62] Kovacs G. Zimmer M. Banko E. Harza I. Antal A. Vidnyanszky Z. 2006 Electrophysiological correlates of visual adaptation to faces and body parts in humans. Cereb. Cortex 16, 742–753 10.1093/cercor/bhj020 (doi:10.1093/cercor/bhj020) 16120795

[63] Winkler C. Rhodes G. 2005 Perceptual adaptation affects attractiveness of female bodies. Br. J. Psychol. 96, 141–154 10.1348/000712605X36343 (doi:10.1348/000712605X36343) 15969827

[64] Hills P. J. Elward R. L. Lewis M. B. 2008 Identity adaptation is mediated and moderated by visualisation ability. Perception 37, 1241–1257 10.1068/p5834 (doi:10.1068/p5834) 18853559

[65] Ryu J. J. Borrmann K. Chaudhuri A. 2008 Imagine Jane and identify John: face identity aftereffects induced by imagined faces. PLoS ONE 3, e2195 10.1371/journal.pone.0002195 (doi:10.1371/journal.pone.0002195) 18493304 PMC2373889

[66] DeBruine L. M. Welling L. L. M. Jones B. C. 2010 Opposite effects of visual versus imagined presentation of faces on subsequent sex perception. Vis. Cogn. 18, 816–828 10.1080/13506281003691357 (doi:10.1080/13506281003691357)

[67] Bestelmeyer P. E. G. Jones B. C. DeBruine L. M. Little A. C. Perrett D. I. Schneider A. Welling L. Conway C. 2008 Sex-contingent face aftereffects depend on perceptual category rather than structural encoding. Cognition 107, 353–365 10.1016/j.cognition.2007.07.018 (doi:10.1016/j.cognition.2007.07.018) 17870064

[68] Rotshtein P. Henson R. N. Treves A. Driver J. Dolan R. J. 2005 Morphing Marilyn into Maggie dissociates physical and identity face representations in the brain. Nat. Neurosci. 8, 107–113 10.1038/nn1370 (doi:10.1038/nn1370) 15592463

[69] Xu X. Yue X. Lescroart M. D. Biederman I. Kim J. G. 2009 Adaptation in the fusiform face area (FFA): image or person? Vis. Res. 49, 2800–2807 10.1016/j.visres.2009.08.021 (doi:10.1016/j.visres.2009.08.021) 19712692

[70] He S. MacLeod D. I. 2001 Orientation-selective adaptation and tilt after-effect from invisible patterns. Nature 411, 473–476 10.1038/35078072 (doi:10.1038/35078072) 11373679

[71] Vul E. MacLeod D. I. 2006 Contingent aftereffects distinguish conscious and preconscious color processing. Nat. Neurosci. 9, 873–874 10.1038/nn1723 (doi:10.1038/nn1723) 16767088

[72] Moradi F. Koch C. Shimojo S. 2005 Face adaptation depends on seeing the face. Neuron 45, 169–175 10.1016/j.neuron.2004.12.018 (doi:10.1016/j.neuron.2004.12.018) 15629711

[73] Vuilleumier P. Armony J. L. Driver J. Dolan R. J. 2001 Effects of attention and emotion on face processing in the human brain: an event-related fMRI study. Neuron 30, 829–841 10.1016/S0896-6273(01)00328-2 (doi:10.1016/S0896-6273(01)00328-2) 11430815

[74] Tamietto M. de Gelder B. 2010 Neural bases of the non-conscious perception of emotional signals. Nat. Rev. Neurosci. 11, 697–709 10.1038/nrn2889 (doi:10.1038/nrn2889) 20811475

[75] Hong S. W. Yang E. Blake R. 2010 Adaptation aftereffects to facial expressions suppressed from visual awareness. J. Vis. 10 (12), 24, 1–13 10.1167/10.12.24 (doi:10.1167/10.12.24) PMC3156693 21047756

[76] Eimer M. Kiss M. Nicholas S. 2010 Response profile of the face-sensitive N170 component: a rapid adaptation study. Cereb. Cortex 20, 2442–2452 10.1093/cercor/bhp312 (doi:10.1093/cercor/bhp312) 20080930

[77] Jacques C. d'Arripe O. Rossion B. 2007 The time course of the inversion effect during individual face discrimination. J. Vis. 7, 1–9 10.1167/7.3.1 (doi:10.1167/7.3.1) 17685810

[78] Kloth N. Schweinberger S. R. Kovacs G. 2010 Neural correlates of generic versus gender-specific face adaptation. J. Cogn. Neurosci. 22, 2345–2356 10.1162/jocn.2009.21329 (doi:10.1162/jocn.2009.21329) 19702459

[79] Harris A. Nakayama K. 2007 Rapid face-selective adaptation of an early extrastriate component in MEG. Cereb. Cortex 17, 63–70 10.1093/cercor/bhj124 (doi:10.1093/cercor/bhj124) 16436684

[80] Harris A. Nakayama K. 2008 Rapid adaptation of the m170 response: importance of face parts. Cereb. Cortex 18, 467–476 10.1093/cercor/bhm078 (doi:10.1093/cercor/bhm078) 17573371

[81] Furl N. van Rijsbergen N. J. Treves A. Friston K. J. Dolan R. J. 2007 Experience-dependent coding of facial expression in superior temporal sulcus. Proc. Natl Acad. Sci. USA 104, 13485–13489 10.1073/pnas.0702548104 (doi:10.1073/pnas.0702548104) 17684100 PMC1948923

[82] Grill-Spector K. Henson R. Martin A. 2006 Repetition and the brain: neural models of stimulus-specific effects. Trends Cogn. Sci. 10, 14–23 10.1016/j.tics.2005.11.006 (doi:10.1016/j.tics.2005.11.006) 16321563

[83] Krekelberg B. Boynton G. M. van Wezel R. J. A. 2006 Adaptation: from single cells to BOLD signals. Trends Neurosci. 29, 250–256 10.1016/j.tins.2006.02.008 (doi:10.1016/j.tins.2006.02.008) 16529826

[84] Fang F. Murray S. O. He S. 2007 Duration-dependent fMRI adaptation and distributed viewer-centered face representation in human visual cortex. Cereb. Cortex 17, 1402–1411 10.1093/cercor/bhl053 (doi:10.1093/cercor/bhl053) 16905593

[85] Furl N. van Rijsbergen N. J. Treves A. Dolan R. J. 2007 Face adaptation aftereffects reveal anterior medial temporal cortex role in high level category representation. Neuroimage 37, 300–310 10.1016/j.neuroimage.2007.04.057 (doi:10.1016/j.neuroimage.2007.04.057) 17561416 PMC2706324

[86] Ng M. Ciaramitaro V. M. Anstis S. Boynton G. M. Fine I. 2006 Selectivity for the configural cues that identify the gender, ethnicity, and identity of faces in human cortex. Proc. Natl Acad. Sci. USA 103, 19 552–19 557 10.1073/pnas.0605358104 (doi:10.1073/pnas.0605358104) PMC1748263 17164335

[87] Loffler G. Yourganov G. Wilkinson F. Wilson H. R. 2005 fMRI evidence for the neural representation of faces. Nat. Neurosci. 8, 1386–1390 10.1038/nn1538 (doi:10.1038/nn1538) 16136037

[88] Winston J. S. Henson R. N. Fine-Goulden M. R. Dolan R. J. 2004 fMRI-adaptation reveals dissociable neural representations of identity and expression in face perception. J. Neurophysiol. 92, 1830–1839 10.1152/jn.00155.2004 (doi:10.1152/jn.00155.2004) 15115795

[89] Andrews T. J. Ewbank M. P. 2004 Distinct representations for facial identity and changeable aspects of faces in the human temporal lobe. Neuroimage 23, 905–913 10.1016/j.neuroimage.2004.07.060 (doi:10.1016/j.neuroimage.2004.07.060) 15528090

[90] Andrews T. J. Davies-Thompson J. Kingstone A. Young A. W. 2010 Internal and external features of the face are represented holistically in face-selective regions of visual cortex. J. Neurosci. 30, 3544–3552 10.1523/JNEUROSCI.4863-09.2010 (doi:10.1523/JNEUROSCI.4863-09.2010) 20203214 PMC2839485

[91] Nishimura M. Doyle J. Humphreys K. Behrmann M. Probing the face-space of individuals with prosopagnosia. Neuropsychologia 48, 1828–1841 10.1016/j.neuropsychologia.2010.03.007 (doi:10.1016/j.neuropsychologia.2010.03.007) 20227431

[92] Susilo T. McKone E. 2010 Impaired face recognition despite normal face-space coding and holistic processing: evidence from a developmental prosopagnosic. J. Vis. 10, 590 10.1167/10.7.590 (doi:10.1167/10.7.590) 22074472

[93] Barrett S. E. O'Toole A. J. 2009 Face adaptation to gender: does adaptation transfer across age categories? Vis. Cogn. 17, 700–715 10.1080/13506280802332197 (doi:10.1080/13506280802332197)

[94] Jaquet E. Rhodes G. 2008 Face aftereffects indicate dissociable, but not distinct, coding of male and female faces. J. Exp. Psychol. Human 34, 101–112 10.1037/0096-1523.34.1.101 (doi:10.1037/0096-1523.34.1.101) 18248142

[95] Jaquet E. Rhodes G. Hayward W. G. 2008 Race-contingent aftereffects suggest distinct perceptual norms for different race faces. Vis. Cogn. 16, 734–753 10.1080/13506280701350647 (doi:10.1080/13506280701350647)

[96] Little A. C. DeBruine L. M. Jones B. C. 2005 Sex-contingent face after-effects suggest distinct neural populations code male and female faces. Proc. R. Soc. B 272, 2283–2287 10.1098/rspb.2005.3220 (doi:10.1098/rspb.2005.3220) PMC1560190 16191641

[97] Little A. C. DeBruine L. M. Jones B. C. Waitt C. 2008 Category contingent aftereffects for faces of different races, ages and species. Cognition 106, 1537–1547 10.1016/j.cognition.2007.06.008 (doi:10.1016/j.cognition.2007.06.008) 17707364

[98] Bestelmeyer P. E. G. Jones B. C. DeBruine L. M. Little A. C. Welling L. L. M. 2009 Face aftereffects suggest interdependent processing of expression and sex and of expression and race. Vis. Cogn. 18, 255–274 10.1080/13506280802708024 (doi:10.1080/13506280802708024)

[99] Fox C. J. Oruc I. Barton J. J. 2008 It doesn't matter how you feel. The facial identity aftereffect is invariant to changes in facial expression. J. Vis. 8, 11–13 10.1167/8.3.11 (doi:10.1167/8.3.11) 18484817

[100] Benton C. P. Etchells P. J. Porter G. Clark A. P. Penton-Voak I. S. Nikolov S. G. 2007 Turning the other cheek: the viewpoint dependence of facial expression after-effects. Proc. R. Soc. B 274, 2131–2137 10.1098/rspb.2007.0473 (doi:10.1098/rspb.2007.0473) PMC2706192 17580295

[101] Benton C. P. Jennings S. J. Chatting D. J. 2006 Viewpoint dependence in adaptation to facial identity. Vis. Res. 46, 3313–3325 10.1016/j.visres.2006.06.002 (doi:10.1016/j.visres.2006.06.002) 16844181

[102] Burke D. Taubert J. Higman T. 2007 Are face representations viewpoint dependent? A stereo advantage for generalising across different views of faces. Vis. Res. 47, 2164–2169 10.1016/j.visres.2007.04.018 (doi:10.1016/j.visres.2007.04.018) 17572467

[103] Jeffery L. Rhodes G. Busey T. 2006 View-specific coding of face shape. Psychol. Sci. 17, 501–505 10.1111/j.1467-9280.2006.01735.x (doi:10.1111/j.1467-9280.2006.01735.x) 16771800

[104] Jeffery L. Rhodes G. Busey T. 2007 Broadly tuned, view-specific coding of face shape: opposing figural aftereffects can be induced in different views. Vis. Res. 47, 3070–3077 10.1016/j.visres.2007.08.018 (doi:10.1016/j.visres.2007.08.018) 17920099

[105] Fang F. He S. 2005 Viewer-centered object representation in the human visual system revealed by viewpoint aftereffects. Neuron 45, 793–800 10.1016/j.neuron.2005.01.037 (doi:10.1016/j.neuron.2005.01.037) 15748853

[106] Bi T. Su J. Chen J. Fang F. 2009 The role of gaze direction in face viewpoint aftereffect. Vis. Res. 49, 2322–2327 10.1016/j.visres.2009.07.002 (doi:10.1016/j.visres.2009.07.002) 19607854

[107] McCollough-Howard C. Webster M. A. 2009 McCollough effect. Scholarpedia: encyclopedia of computational neuroscience. See http://www.scholarpedia.org/article/McCollough_effect

[108] Durgin F. H. 1996 Visual aftereffect of texture density contingent on color of frame. Percept. Psychophys. 58, 207–223 10.3758/BF03211876 (doi:10.3758/BF03211876) 8838165

[109] Treisman A. M. Gelade G. 1980 A feature-integration theory of attention. Cogn. Psychol. 12, 97–136 10.1016/0010-0285(80)90005-5 (doi:10.1016/0010-0285(80)90005-5) 7351125

[110] Lee K. Byatt G. Rhodes G. 2000 Caricature effects, distinctiveness, and identification: testing the face-space framework. Psychol. Sci. 11, 379–385 10.1111/1467-9280.00274 (doi:10.1111/1467-9280.00274) 11228908

[111] Rhodes G. McLean I. G. 1990 Distinctiveness and expertise effects with homogeneous stimuli: towards a model of configural coding. Perception 19, 773–794 10.1068/p190773 (doi:10.1068/p190773) 2130375

[112] Rhodes G. Brennan S. Carey S. 1987 Identification and ratings of caricatures: implications for mental representations of faces. Cogn. Psychol. 19, 473–497 10.1016/0010-0285(87)90016-8 (doi:10.1016/0010-0285(87)90016-8) 3677584

[113] Leopold D. A. Bondar I. V. Giese M. A. 2006 Norm-based face encoding by single neurons in the monkey inferotemporal cortex. Nature 442, 572–575 10.1038/nature04951 (doi:10.1038/nature04951) 16862123

[114] Nosofsky R. M. 1986 Attention, similarity, and the identification-categorization relationship. J. Exp. Psychol. Gen. 115, 39–61 10.1037/0096-3445.115.1.39 (doi:10.1037/0096-3445.115.1.39) 2937873

[115] Valentine T. Endo M. 1992 Towards an exemplar model of face processing: the effects of race and distinctiveness. Q. J. Exp. Psychol. A 44, 671–703 1615169 10.1080/14640749208401305

[116] Blakemore C. Campbell F. W. 1969 On the existence of neurones in the human visual system selectively sensitive to the orientation and size of retinal images. J. Physiol. 203, 237–260 5821879 10.1113/jphysiol.1969.sp008862 PMC1351526

[117] Calder A. J. Jenkins R. Cassel A. Clifford C. W. G. 2008 Visual representation of eye gaze is coded by a nonopponent multichannel system. J. Exp. Psychol. Gen. 137, 244–261 10.1037/0096-3445.137.2.244 (doi:10.1037/0096-3445.137.2.244) 18473657

[118] Jenkins R. Beaver J. D. Calder A. J. 2006 I thought you were looking at me: direction-specific aftereffects in gaze perception. Psychol. Sci. 17, 506–513 10.1111/j.1467-9280.2006.01736.x (doi:10.1111/j.1467-9280.2006.01736.x) 16771801

[119] Webster M. A. Leonard D. 2008 Adaptation and perceptual norms in color vision. J. Optic. Soc. Am. A 25, 2817–2825 10.1364/JOSAA.25.002817 (doi:10.1364/JOSAA.25.002817) PMC2657039 18978861

[120] Eisner A. Macleod D. I. 1981 Flicker photometric study of chromatic adaption: selective suppression of cone inputs by colored backgrounds. J. Optic. Soc. Am. 71, 705–717 10.1364/JOSA.71.000705 (doi:10.1364/JOSA.71.000705) 7252613

[121] Robbins R. McKone E. Edwards M. 2007 Aftereffects for face attributes with different natural variability: adapter position effects and neural models. J. Exp. Psychol. Human 33, 570–592 10.1037/0096-1523.33.3.570 (doi:10.1037/0096-1523.33.3.570) 17563222

[122] Rhodes G. Jeffery L. 2006 Adaptive norm-based coding of facial identity. Vis. Res. 46, 2977–2987 10.1016/j.visres.2006.03.002 (doi:10.1016/j.visres.2006.03.002) 16647736

[123] Tsao D. Y. Freiwald W. A. 2006 What's so special about the average face? Trends Cogn. Sci. 10, 391–393 10.1016/j.tics.2006.07.009 (doi:10.1016/j.tics.2006.07.009) 16899396

[124] Benton C. P. Burgess E. C. 2008 The direction of measured face aftereffects. J. Vis. 8, 1–6 10.1167/8.15.1 (doi:10.1167/8.15.1) 19146285

[125] Regan D. Hamstra S. J. 1992 Shape discrimination and the judgement of perfect symmetry: dissociation of shape from size. Vis. Res. 32, 1845–1864 10.1016/0042-6989(92)90046-L (doi:10.1016/0042-6989(92)90046-L) 1287983

[126] Suzuki S. 2005 High-level pattern coding revealed by brief shape aftereffects. In Fitting the mind to the world: adaptation and aftereffects in high-level vision. *Advances in visual cognition series* (eds Clifford C. Rhodes G.), vol. 2, pp. 138–172 Oxford, UK: Oxford University Press

[127] Blakemore C. Sutton P. 1969 Size adaptation: a new aftereffect. Science 166, 245–247 10.1126/science.166.3902.245 (doi:10.1126/science.166.3902.245) 5809598

[128] Field D. J. Brady N. 1997 Visual sensitivity, blur and the sources of variability in the amplitude spectra of natural scenes. Vis. Res. 37, 3367–3383 10.1016/S0042-6989(97)00181-8 (doi:10.1016/S0042-6989(97)00181-8) 9425550

[129] Webster M. A. Georgeson M. A. Webster S. M. 2002 Neural adjustments to image blur. Nat. Neurosci. 5, 839–840 10.1038/nn906 (doi:10.1038/nn906) 12195427

[130] MacLeod D. I. A. von der Twer T. 2003 The pleistochrome: optimal opponent codes for natural colours. In Colour perception: from light to object (eds Heyer D. Mausfeld R.), pp. 155–184. London, UK: Oxford University Press

[131] Yuille A. L. Grzywacz N. M. 1989 A winner-take-all mechanism based on presynaptic inhibition feedback. Neural Comput. 1, 334–347 10.1162/neco.1989.1.3.334 (doi:10.1162/neco.1989.1.3.334)

[132] Nishimura M. Maurer D. Jeffery L. Pellicano E. Rhodes G. 2008 Fitting the child's mind to the world: adaptive norm-based coding of facial identity in 8-year-olds. Dev. Sci. 11, 620–627 10.1111/j.1467-7687.2008.00706.x (doi:10.1111/j.1467-7687.2008.00706.x) 18576969

[133] Dacey D. M. Lee B. B. Stafford D. K. Pokorny J. Smith V. C. 1996 Horizontal cells of the primate retina: cone specificity without spectral opponency. Science 271, 656–659 10.1126/science.271.5249.656 (doi:10.1126/science.271.5249.656) 8571130

[134] He S. MacLeod D. I. A. 1997 Contrast-modulation flicker: dynamics and spatial resolution of the light adaptation process. Vis. Res. 38, 985–1000 10.1016/S0042-6989(97)00290-3 (doi:10.1016/S0042-6989(97)00290-3) 9666981

[135] MacLeod D. I. A. Williams D. R. Makous W. 1992 A visual nonlinearity fed by single cones. Vis. Res. 32, 347–363 10.1016/0042-6989(92)90144-8 (doi:10.1016/0042-6989(92)90144-8) 1574850

[136] Southall J. P. C. (ed.) 1962 Helmholtz's treatise on physiological optics. New York, NY: Dover

[137] MacLeod D. I. 1974 Directionally selective light adaptation: a visual consequence of receptor disarray? Vis. Res. 14, 369–378 10.1016/0042-6989(74)90235-1 (doi:10.1016/0042-6989(74)90235-1) 4851396

[138] Mollon J. D. 1982 Color vision. Ann. Rev. Psychol. 33, 41–85 10.1146/annurev.ps.33.020182.000353 (doi:10.1146/annurev.ps.33.020182.000353) 6977310

[139] Krauskopf J. Williams D. R. Heeley D. W. 1982 Cardinal directions of color space. Vis. Res. 22, 1123–1131 10.1016/0042-6989(82)90077-3 (doi:10.1016/0042-6989(82)90077-3) 7147723

[140] Webster M. A. Mollon J. D. 1994 The influence of contrast adaptation on color appearance. Vis. Res. 34, 1993–2020 10.1016/0042-6989(94)90028-0 (doi:10.1016/0042-6989(94)90028-0) 7941399

[141] Spetch M. L. Cheng K. Clifford C. W. G. 2004 Peak shift but not range effects in recognition of faces. Learning Motivation 35, 221–241 10.1016/j.lmot.2003.11.001 (doi:10.1016/j.lmot.2003.11.001)

[142] Muskat J. A. Paras C. L. Webster M. A. 2000 Adaptation to collections of faces. Invest. Ophth. 41, S222

[143] Webster M. A. Mollon J. D. 1991 Changes in colour appearance following post-receptoral adaptation. Nature 349, 235–238 10.1038/349235a0 (doi:10.1038/349235a0) 1987475

[144] Webster M. A. Wilson J. A. 2000 Interactions between chromatic adaptation and contrast adaptation in color appearance. Vis. Res. 40, 3801–3816 10.1016/S0042-6989(00)00238-8 (doi:10.1016/S0042-6989(00)00238-8) 11090672

[145] Barlow H. B. 1990 A theory about the functional role and synaptic mechanism of visual aftereffects. In Visual coding and efficiency (ed. Blakemore C.), pp. 363–375 Cambridge, UK: Cambridge University Press

[146] Russell R. 2009 A sex difference in facial contrast and its exaggeration by cosmetics. Perception 38, 1211–1219 10.1068/p6331 (doi:10.1068/p6331) 19817153

[147] Rolls E. T. Baylis G. C. 1986 Size and contrast have only small effects on the responses to faces of neurons in the cortex of the superior temporal sulcus of the monkey. Exp. Brain Res. 65, 38–48 10.1007/BF00243828 (doi:10.1007/BF00243828) 3803509

[148] Avidan G. Harel M. Hendler T. Ben-Bashat D. Zohary E. Malach R. 2002 Contrast sensitivity in human visual areas and its relationship to object recognition. J. Neurophysiol. 87, 3102–3116 12037211 10.1152/jn.2002.87.6.3102

[149] Anderson N. D. Habak C. Wilkinson F. Wilson H. R. 2007 Evaluating shape after-effects with radial frequency patterns. Vis. Res. 47, 298–308 10.1016/j.visres.2006.02.013 (doi:10.1016/j.visres.2006.02.013) 17178143

[150] Suzuki S. 2001 Attention-dependent brief adaptation to contour orientation: a high-level aftereffect for convexity? Vis. Res. 41, 3883–3902 10.1016/S0042-6989(01)00249-8 (doi:10.1016/S0042-6989(01)00249-8) 11738454

[151] Oruc I. Barton J. J. 2010 A novel face aftereffect based on recognition contrast thresholds. Vis. Res. 50, 1845–1854 10.1016/j.visres.2010.06.005 (doi:10.1016/j.visres.2010.06.005) 20558191

[152] Werner J. S. Schefrin B. E. 1993 Loci of achromatic points throughout the life span. J. Optic. Soc. Am. A 10, 1509–1516 10.1364/JOSAA.10.001509 (doi:10.1364/JOSAA.10.001509) 8350147

[153] Kay P. Regier T. 2006 Language, thought and color: recent developments. Trends Cogn. Sci. 10, 51–54 10.1016/j.tics.2005.12.007 (doi:10.1016/j.tics.2005.12.007) 16386939

[154] Rhodes G. 2006 The evolutionary psychology of facial beauty. Ann. Rev. Psychol. 57, 199–226 10.1146/annurev.psych.57.102904.190208 (doi:10.1146/annurev.psych.57.102904.190208) 16318594

[155] Webster M. A. Yasuda M. Haber S. Ballardini N. Leonard D. 2007 Adaptation and perceptual norms. In Human vision and electronic imaging (eds Rogowitz B. E. Pappas T. N.), *Proc. SPIE* **6492**, 1–11 10.1117/12.730399 (doi:10.1117/12.730399)

[156] Walraven J. Werner J. S. 1991 The invariance of unique white; a possible implication for normalizing cone action spectra. Vis. Res. 31, 2185–2193 10.1016/0042-6989(91)90171-Z (doi:10.1016/0042-6989(91)90171-Z) 1771798

[157] Werner J. S. Walraven J. 1982 Effect of chromatic adaptation on the achromatic locus: the role of contrast, luminance and background color. Vis. Res. 22, 929–943 10.1016/0042-6989(82)90029-3 (doi:10.1016/0042-6989(82)90029-3) 7135855

[158] Bartlett M. S. 2007 Information maximization in face processing. Neurocomputing 70, 2204–2217 10.1016/j.neucom.2006.02.025 (doi:10.1016/j.neucom.2006.02.025)

[159] MacLeod D. I. A. 2003 Colour discrimination, colour constancy, and natural scene statistics (The Verriest Lecture). In Normal and defective colour vision (eds Mollon J. D. Pokorny J. Knoblauch K.), pp. 189–218 London, UK: Oxford University Press

[160] Burton A. M. Vokey J. R. 1998 The face-space typicality paradox: understanding the face-space metaphor. Q. J. Exp. Psychol. A: Hum. Exp. Psychol. 51, 475–483

[161] Le Grande Y. 1949 Les seuils differentiells de couleurs dans la theorie de Young. Rev. Opt. 28, 261–278 18151607

[162] Pugh E. N. Jr Mollon J. D. 1979 A theory of the pi1 and pi3 color mechanisms of Stiles. Vis. Res. 19, 293–312 10.1016/0042-6989(79)90175-5 (doi:10.1016/0042-6989(79)90175-5) 444331

[163] Friele L. F. C. 1961 Analysis of the Brown and Brown-MacAdam colour discrimination data. Die Farbe 10, 193–224

[164] Nachmias J. 2008 Judging spatial properties of simple figures. Vis. Res. 48, 1290–1296 10.1016/j.visres.2008.02.024 (doi:10.1016/j.visres.2008.02.024) 18407313

[165] Chaparro A. Stromeyer C. F. 3rd Huang E. P. Kronauer R. E. Eskew R. T. Jr 1993 Colour is what the eye sees best. Nature 361, 348–350 10.1038/361348a0 (doi:10.1038/361348a0) 8426653

[166] Webster M. A. Mollon J. D. 1997 Adaptation and the color statistics of natural images. Vis. Res. 37, 3283–3298 10.1016/S0042-6989(97)00125-9 (doi:10.1016/S0042-6989(97)00125-9) 9425544

[167] Thornton J. E. Pugh E. N. Jr 1983 Red/green color opponency at detection threshold. Science 219, 191–193 10.1126/science.6849131 (doi:10.1126/science.6849131) 6849131

[168] Whittle P. 1992 Brightness, discriminability and the ‘crispening effect’. Vis. Res. 32, 1493–1507 10.1016/0042-6989(92)90205-W (doi:10.1016/0042-6989(92)90205-W) 1455722

[169] Laughlin S. B. 1983 Matching coding to scenes to enhance efficiency. In Biological processing of images (eds Braddick O. J. Sleigh A. C.), pp. 42–52 Berlin, Germany: Springer

[170] Regan B. C. Julliot C. Simmen B. Vienot F. Charles-Dominique P. Mollon J. D. 2001 Fruits, foliage and the evolution of primate colour vision. Phil. Trans. R. Soc. Lond. B 356, 229–283 10.1098/rstb.2000.0773 (doi:10.1098/rstb.2000.0773) 11316480 PMC1088428

[171] Webster M. A. 1996 Human colour perception and its adaptation. Network-Comp. Neural 7, 587–634 10.1088/0954-898X/7/4/002 (doi:10.1088/0954-898X/7/4/002)

[172] Lennie P. Movshon J. A. 2005 Coding of color and form in the geniculostriate visual pathway. J. Optic. Soc. Am. A 22, 2013–2033 10.1364/JOSAA.22.002013 (doi:10.1364/JOSAA.22.002013) 16277273

[173] Zaidi Q. Shapiro A. G. 1993 Adaptive orthogonalization of opponent-color signals. Biol. Cybernet. 69, 415–428 8274540

[174] Atick J. J. Li Z. Redlich A. N. 1993 What does post-adaptation color appearance reveal about cortical color representation? Vis. Res. 33, 123–129 10.1016/0042-6989(93)90065-5 (doi:10.1016/0042-6989(93)90065-5) 8451837

[175] Hurvich L. M. Jameson D. 1957 An opponent-process theory of color vision. Psychol. Rev. 64, 384–404 10.1037/h0041403 (doi:10.1037/h0041403) 13505974

[176] Derrington A. M. Krauskopf J. Lennie P. 1984 Chromatic mechanisms in lateral geniculate nucleus of macaque. J. Physiol. 357, 241–265 6512691 10.1113/jphysiol.1984.sp015499 PMC1193257

[177] De Valois R. L. De Valois K. K. 1993 A multi-stage color model. Vis. Res. 33, 1053–1065 10.1016/0042-6989(93)90240-W (doi:10.1016/0042-6989(93)90240-W) 8506645

[178] Mollon J. D. 2006 Monge (The Verriest Lecture). Vis. Neurosci. 23, 297–309 10.1017/S0952523806233479 (doi:10.1017/S0952523806233479) 16961961

[179] Stoughton C. M. Conway B. R. 2008 Neural basis for unique hues. Curr. Biol. 18, R698–R699 10.1016/j.cub.2008.06.018 (doi:10.1016/j.cub.2008.06.018) 18727902

[180] Mollon J. D. 2009 A neural basis for unique hues? Curr. Biol. 19, R441–R442 10.1016/j.cub.2009.05.008 (doi:10.1016/j.cub.2009.05.008) 19515347

[181] Ekman P. 1992 Facial expressions of emotion: an old controversy and new findings. Phil. Trans. R. Soc. Lond. B 335, 63–69 10.1098/rstb.1992.0008 (doi:10.1098/rstb.1992.0008) 1348139

[182] Ekman P. 1992 An argument for basic emotions. Cogn. Emotion 6, 169–200 10.1080/02699939208411068 (doi:10.1080/02699939208411068)

[183] Plutchik R. 2001 The nature of emotions. Am. Scient. 89, 344–350

[184] Rutherford M. D. Chattha H. M. Krysko K. M. 2008 The use of aftereffects in the study of relationships among emotion categories. J. Exp. Psychol. Human 34, 27–40 10.1037/0096-1523.34.1.27 (doi:10.1037/0096-1523.34.1.27) 18248138

[185] Juricevic I. Webster M. 2008 Adaptation to facial expressions. J. Vis. 8, 712a 10.1167/8.6.712 (doi:10.1167/8.6.712)

[186] Smith M. L. Cottrell G. W. Gosselin F. Schyns P. G. 2005 Transmitting and decoding facial expressions. Psychol. Sci. 16, 184–189 10.1111/j.0956-7976.2005.00801.x (doi:10.1111/j.0956-7976.2005.00801.x) 15733197

[187] Calder A. J. Young A. W. 2005 Understanding the recognition of facial identity and facial expression. Nat. Rev. Neurosci. 6, 641–651 10.1038/nrn1724 (doi:10.1038/nrn1724) 16062171

[188] Wilson H. R. Loffler G. Wilkinson F. 2002 Synthetic faces, face cubes, and the geometry of face space. Vis. Res. 42, 2909–2923 10.1016/S0042-6989(02)00362-0 (doi:10.1016/S0042-6989(02)00362-0) 12450502

[189] Tanaka J. Giles M. Kremen S. Simon V. 1998 Mapping attractor fields in face space: the atypicality bias in face recognition. Cognition 68, 199–220 10.1016/S0010-0277(98)00048-1 (doi:10.1016/S0010-0277(98)00048-1) 9852665

[190] Tanaka J. W. Corneille O. 2007 Typicality effects in face and object perception: further evidence for the attractor field model. Percept. Psychophys. 69, 619–627 10.3758/BF03193919 (doi:10.3758/BF03193919) 17727115

[191] Dakin S. C. Omigie D. 2009 Psychophysical evidence for a non-linear representation of facial identity. Vis. Res. 49, 2285–2296 10.1016/j.visres.2009.06.016 (doi:10.1016/j.visres.2009.06.016) 19555705 PMC2741567

[192] Rhodes G. Maloney L. T. Turner J. Ewing L. 2007 Adaptive face coding and discrimination around the average face. Vis. Res. 47, 974–989 10.1016/j.visres.2006.12.010 (doi:10.1016/j.visres.2006.12.010) 17316740

[193] Rhodes G. Watson T. L. Jeffery L. Clifford C. W. 2010 Perceptual adaptation helps us identify faces. Vis. Res. 50, 963–968 10.1016/j.visres.2010.03.003 (doi:10.1016/j.visres.2010.03.003) 20214920

[194] Ng M. Boynton G. M. Fine I. 2008 Face adaptation does not improve performance on search or discrimination tasks. J. Vis. 8, 1–20 10.1167/8.1.1 (doi:10.1167/8.1.1) 18318604 PMC3175108

[195] Clifford C. W. Webster M. A. Stanley G. B. Stocker A. A. Kohn A. Sharpee T. O. Schwartz O. 2007 Visual adaptation: neural, psychological and computational aspects. Vis. Res. 47, 3125–3131 17936871 10.1016/j.visres.2007.08.023

[196] Barlow H. B. Macleod D. I. van Meeteren A. 1976 Adaptation to gratings: no compensatory advantages found. Vis. Res. 16, 1043–1045 10.1016/0042-6989(76)90241-8 (doi:10.1016/0042-6989(76)90241-8) 969214

[197] Westheimer G. Gee A. 2002 Orthogonal adaptation and orientation discrimination. Vis. Res. 42, 2339–2343 10.1016/S0042-6989(02)00192-X (doi:10.1016/S0042-6989(02)00192-X) 12350422

[198] Clifford C. W. G. Wyatt A. M. Arnold D. H. Smith S. T. Wenderoth P. 2001 Orthogonal adaptation improves orientation discrimination. Vis. Res. 41, 151–159 10.1016/S0042-6989(00)00248-0 (doi:10.1016/S0042-6989(00)00248-0) 11163850

[199] Regan D. Beverley K. I. 1985 Postadaptation orientation discrimination. J. Optic. Soc. Am. A 2, 147–155 10.1364/JOSAA.2.000147 (doi:10.1364/JOSAA.2.000147) 3973752

[200] Snippe H. Koenderink J. 1992 Discrimination thresholds for channel-coded systems. Biol. Cybernet. 66, 543–551 10.1007/BF00204120 (doi:10.1007/BF00204120) 1627687

[201] Pallett P. M. MacLeod D. I. A. 2011 Seeing faces as objects: no face inversion effect with geometrical discrimination. Attention Percept. Psychophys. 73, 504–520 10.3758/S13414-010-0033-2 (doi:10.3758/S13414-010-0033-2) PMC3037484 21264731

[202] Shevell S. K. Kingdom F. A. 2008 Color in complex scenes. Ann. Rev. Psychol. 59, 143–166 10.1146/annurev.psych.59.103006.093619 (doi:10.1146/annurev.psych.59.103006.093619) 18154500

[203] Webster M. A. Werner J. S. Field D. J. 2005 Adaptation and the phenomenology of perception. In Fitting the mind to the world: adaptation and aftereffects in high-level vision. *Advances in visual cognition series* (eds Clifford C. Rhodes G.), vol. 2, pp. 241–277 Oxford, UK: Oxford University Press

[204] Beer D. Wortman J. Horwitz G. MacLeod D. 2005 Compensation of white for macular filtering. J. Vis. 5, 282a 10.1167/5.8.282 (doi:10.1167/5.8.282)

[205] Delahunt P. B. Webster M. A. Ma L. Werner J. S. 2004 Long-term renormalization of chromatic mechanisms following cataract surgery. Vis. Neurosci. 21, 301–307 10.1017/S0952523804213025 (doi:10.1017/S0952523804213025) 15518204 PMC2633455

[206] Webster M. A. Halen K. Meyers A. J. Winkler P. Werner J. S. 2010 Colour appearance and compensation in the near periphery. Proc. R. Soc. B 277, 1817–1825 10.1098/rspb.2009.1832 (doi:10.1098/rspb.2009.1832) PMC2871866 20147325

[207] Pellicano E. Jeffery L. Burr D. Rhodes G. 2007 Abnormal adaptive face-coding mechanisms in children with autism spectrum disorder. Curr. Biol. 17, 1508–1512 10.1016/j.cub.2007.07.065 (doi:10.1016/j.cub.2007.07.065) 17764946

[208] Saxton T. K. Little A. C. DeBruine L. M. Jones B. C. Roberts S. C. 2009 Adolescents' preferences for sexual dimorphism are influenced by relative exposure to male and female faces. Pers. Indiv. Differ. 47, 864–868 10.1016/j.paid.2009.07.005 (doi:10.1016/j.paid.2009.07.005)

[209] Apicella C. L. Little A. C. Marlowe F. W. 2007 Facial averageness and attractiveness in an isolated population of hunter–gatherers. Perception 36, 1813–1820 10.1068/p5601 (doi:10.1068/p5601) 18283931

[210] Tovee M. J. Swami V. Furnham A. Mangalparsad R. 2006 Changing perceptions of attractiveness as observers are exposed to a different culture. Evol. Hum. Behav. 27, 4430456 10.1016/j.evolhumbehav.2006.05.004 (doi:10.1016/j.evolhumbehav.2006.05.004)

[211] Malinowski 1929 The sexual life of savages in north-western Melanesia. London, UK: Routledge and Kegan Paul

[212] Atick J. J. Griffin P. A. Redlich A. N. 1996 The vocabulary of shape: principal shapes for probing perception and neural response. Network-Comp. Neural 7, 1–5 10.1088/0954-898X/7/1/002 (doi:10.1088/0954-898X/7/1/002) 29480143

[213] Nestor A. Tarr M. J. 2008 Gender recognition of human faces using color. Psychol. Sci. 19, 1242–1246 10.1111/j.1467-9280.2008.02232.x (doi:10.1111/j.1467-9280.2008.02232.x) 19121131

[214] Jones B. C. Little A. C. Burt D. M. Perrett D. I. 2004 When facial attractiveness is only skin deep. Perception 33, 569–576 10.1068/p3463 (doi:10.1068/p3463) 15250662

[215] Changizi M. A. Zhang Q. Shimojo S. 2006 Bare skin, blood and the evolution of primate colour vision. Biol. Lett. 2, 217–221 10.1098/rsbl.2006.0440 (doi:10.1098/rsbl.2006.0440) 17148366 PMC1618887

[216] Mollon J. D. 1989 ‘Tho' she kneel'd in that place where they grew…’ The uses and origins of primate colour vision. J. Exp. Biol. 146, 21–38 2689563 10.1242/jeb.146.1.21

[217] Yasuda M. Webster S. M. Webster M. A. 2007 Color and facial expressions. J. Vis. 7, 946a

